# Attention to local and global levels of hierarchical Navon figures affects rapid scene categorization

**DOI:** 10.3389/fpsyg.2014.01274

**Published:** 2014-12-02

**Authors:** John Brand, Aaron P. Johnson

**Affiliations:** ^1^Department of Psychology, Concordia UniversityMontreal, QC, Canada; ^2^Centre for Interdisciplinary Research in Rehabilitation of Greater MontrealMontreal, QC, Canada

**Keywords:** Navon processing, GIST, scene categorization, attention, spatial frequency

## Abstract

In four experiments, we investigated how attention to local and global levels of hierarchical Navon figures affected the selection of diagnostic spatial scale information used in scene categorization. We explored this issue by asking observers to classify hybrid images (i.e., images that contain low spatial frequency (LSF) content of one image, and high spatial frequency (HSF) content from a second image) immediately following global and local Navon tasks. Hybrid images can be classified according to either their LSF, or HSF content; thus, making them ideal for investigating diagnostic spatial scale preference. Although observers were sensitive to both spatial scales (Experiment 1), they overwhelmingly preferred to classify hybrids based on LSF content (Experiment 2). In Experiment 3, we demonstrated that LSF based hybrid categorization was faster following global Navon tasks, suggesting that LSF processing associated with global Navon tasks primed the selection of LSFs in hybrid images. In Experiment 4, replicating Experiment 3 but suppressing the LSF information in Navon letters by contrast balancing the stimuli examined this hypothesis. Similar to Experiment 3, observers preferred to classify hybrids based on LSF content; however and in contrast, LSF based hybrid categorization was slower following global than local Navon tasks.

## Introduction

The ability to perceive a scene under increased attentional load is often cited as evidence of pre-attentive scene perception. This evidence is typically indexed using dual-task paradigms in which a secondary scene categorization task is unaffected by a concurrent, cognitively demanding primary task. Researchers argue that scene perception is pre-attentive as it is immune to inattentional blindness (Mack and Rock, 1998), unimpaired under dual task conditions (Li et al., 2002; Rousselet et al., 2002), susceptible to stroop interference (Greene and Fei-Fei, 2014), and impervious to change blindness if the object's removal does not change the meaning of the scene (Rensink et al., 1997; Simons and Levin, 1997).

However, other researchers question the evidence in support of the automaticity of scene perception. Cohen et al. (2011) argued that previous studies falsely demonstrated pre-attentive scene perception because they failed to use sufficiently demanding primary tasks, thereby allowing attentional resources to be allocated to the scene stimuli. By increasing the primary task difficulty, Cohen and colleagues demonstrated that concurrently completing multiple-object tracking and serial representation visual presentation (RSVP) tasks impairs scene categorization. Together with previous research in which deficits in scene perception were indexed using attentional blink (Marois et al., 2004; Evans and Treisman, 2005; Slagter et al., 2010), inattentional blindness (Mack and Clarke, 2012), and dual task (Walker et al., 2008) paradigms, Cohen and colleagues concluded that conscious scene perception requires attention.

Although concluding that attention is necessary for a scene to reach conscious awareness, Cohen et al. (2011) acknowledged that some higher-level aspects of scene processing occur in the absence of attention. One of the strongest findings in support of this hypothesis is the presence of scene-related behaviors that occur so rapid that attention is thought to play little or no role. Kirchner and Thorpe's (2006; see also, Crouzet et al., 2010) study illustrates this point. They showed that when two natural images are presented concurrently, observers are able to make an ultra-rapid saccade to the image that contained an animal in as little as 120–130 ms. Consistent with this view, Thorpe et al. (1996; see also, Fabre-Thorpe et al., 2001) showed that observers are able to remove their finger from a button box within 300 ms in response to the presence of an animal. Critically, simultaneous event-related potentials revealed a differential frontal lobe activity between target and non-target displays approximately 150 ms after stimulus onset. This suggests that scene categorization is made prior to this time point. Researchers (VanRullen and Thorpe, 2002) cite such results as evidence that scene categorization is accomplished, in part, by an automatic feed-forward mechanism, a conclusion corroborated by simulation evidence (Serre et al., 2007).

The rapid ability to categorize scenes suggests that a scene's semantic content is based on information originating from early visual processes. Consistent with this idea, Schyns and Oliva (1994) suggested that rapid scene categorization is based on a scene's global layout. Highways, for example, tend to have fewer vertical straight lines compared to city landscapes that have many dense, vertical orientations. Although these global image properties can vary from one scene to another (e.g., some cities are less dense than others), the consistency of spatial organization across different scenes is thought to activate a scene schema that can be used for rapid scene categorization. Schyns and Oliva tested this hypothesis by introducing a new type of scene stimuli, termed a hybrid image. A hybrid image contains information from two separate sources at different spatial frequencies. For example, an image that contains the low spatial frequency (LSF) content of one picture (e.g., a city scene), and the high spatial frequency (HSF) content of a second picture (e.g., a highway scene). Of particular importance to Schyns and Oliva was not spatial frequency *per se*, but rather the information that each spatial scale conveyed for scene recognition. Converging evidence from neurophysiological and psychophysical studies suggest that visual information is organized into spatial frequency channels in which global information is conveyed by LSFs and finer information is conveyed by HSFs (for a review, see Morrison and Schyns, 2001). Consequently, the authors reasoned that if scene recognition is based on coarse information, then observers should prefer to categorize hybrid images based on LSF content.

To test their hypothesis, Schyns and Oliva (1994) asked observers to indicate whether a briefly presented (30 or 150 ms) sample image matched a subsequent target image. The sample image was either a hybrid, low-pass filtered (i.e., contained only LSFs), high-pass filtered (i.e., contained only HSFs), or a full broadband spatial frequency scene (i.e., an unaltered original image). The target image was always a broadband image. Of critical importance here was the association between hybrid samples and target images. On LSF-hybrid trials, the hybrid's LSF content matched the target scene. On HSF-hybrid trials, the hybrid's HSF content matched the target scene. When presentation duration was short, LSF-hybrid trials were more accurate than HSF-hybrid trials; conversely, when presentation duration was long, HSF-hybrid trials were more accurate than LSF-hybrid trials. Critically, categorization performance was high for all control conditions, suggesting that differences in spatial frequency availability cannot account for the differential processing of hybrid images. Schyns and Oliva attributed this result to a coarse-to-fine processing bias in which the early availability of a scene's global layout activates a scene schema from memory. Finer details emerge later and fill in the details of the scene's content (e.g., object recognition).

Oliva and Schyns (1997) modified the coarse-to-fine hypothesis to reflect the fact that either global, or fine scale information can be used for scene recognition. They asked observers to first complete a sensitization phase during which they were briefly presented natural images that were meaningful at only one spatial frequency (e.g., a LSF version of a highway scene with HSF structured noise). A test phase immediately followed in which observers were asked to classify hybrid images. Observers were more likely to categorize hybrids based on LSF and HSF content, respectively, if they were first sensitized to the same frequencies during the sensitization phase. Interestingly, observers claimed to be aware of only a single spatial scale within the hybrid images, suggesting that diagnostic scale selection was based on the scale that was previously the most informative.

To explain this flexibility in spatial scale selection, Oliva and Schyns (1997) suggested that attention is driven to diagnostic spatial frequencies in which recognition is based on scale specific cues of a scene category (e.g., natural landscapes contain LSFs at a horizontal orientation that correspond to the horizon). This idea dovetails with Chong and Treisman's (2005) notion that different distributions of attention facilitate the extraction of different types of information within a scene. According to Chong and Treisman, a scene's layout is organized hierarchically and attention can be deployed either locally, globally, or distributed over a set of similar items. When attention is focused locally, features are bound together resulting in the identification of an object. In contrast, when attention is distributed globally, the gist or semantic meaning of a scene is extracted based on its global layout. Finally, when attention is distributed over a set of similar items, summary representations of set properties are automatically extracted (e.g., average size; Ariely, 2001).

Global and local distributions of attention are typically studied using hierarchical Navon stimuli (e.g., a large “A” comprised of smaller “Cs”). Navon (1977) reported a global precedence effect that is characterized by two robust findings. First, global letters are identified faster than local letters; and second, global recognition interferes with local recognition but not vice versa. Several researchers (Shulman and Wilson, 1987; Badcock et al., 1990) explained the global precedence effect using the coarse-to-fine processing framework. Similar to the identification of coarse and fine information, the hypothesis is that the identification of global and local information is based on LSF and HSF information, respectively. In addition, Flevaris et al. (2011) showed that adopting different attentional distributions facilitates the selection of different spatial scales. They asked participants to classify the orientation of either the LSF or HSF component of a compound sine-wave grating immediately following global, or local Navon tasks. When discriminating the orientation of the LSF component, observers were faster following global Navon tasks; conversely, when asked to discriminate the orientation of the HSF component, observers were faster following local Navon tasks.

Flevaris et al.'s (2011) result suggests that attending to global and local levels should differentially affect scene categorization by facilitating the selection of LSFs and HSFs, respectively. In the present research, we tested this hypothesis by asking participants to categorize briefly presented hybrid images following global, or local Navon tasks. However, because hybrid images contain competing sources of categorization content, it was important that we first demonstrated the ability of our observers to extract both sources of information. Additionally, it was also important that we understood the spatial frequency that our observers preferred to use for categorization, irrespective of any attention manipulation. Thus, in Experiment 1 we assessed spatial scale sensitivity and in Experiment 2 we assessed diagnostic spatial scale preference.

Experiment 1 was a probe design similar to Schyns and Oliva (1994) in which observers were asked to indicate whether a probe word matched a briefly presented (32 or 150 ms) hybrid image. The probe word matched either the hybrid's LSF, or HSF content. In a control condition, the probe word matched neither spatial frequency. The measure, *d* prime (*d*′) was computed to measure observers' sensitivity to both LSFs, and HSFs. *d*′ values were above 1.5 in each condition, suggesting that both LSFs and HSFs are available in our hybrid images, at both short and long durations. Experiment 2 was a replication of Experiment 1, with the exception that we used an all-alternative forced choice paradigm in which observers were asked to choose the image category from a list of all possible target categories. Critically, this design allowed us to compute an objective measure of preferred diagnostic spatial scale. Results indicated that observers preferred to categorize hybrid images based on LSF content, at both short and long durations. Together with the results of Experiment 1, Experiment 2 demonstrated that our observers preferred to base categorization on LSF content, despite the fact that both LSFs, and HSFs were perceptually available.

The fact that our observers preferred to base hybrid categorization on LSF content suggests that attending globally facilitates scene categorization. A consequence of this prediction is that LSF-based hybrid categorization should be faster following global compared to local Navon tasks. In Experiment 3, we directly tested this hypothesis by asking observers to classify hybrid images immediately following global and local Navon tasks. Similar to Experiment 2, observers preferred to categorize hybrid images based on LSF content. Furthermore, and consistent with our hypothesis, LSF-based hybrid image categorization was faster following global Navon tasks. In Experiment 4, we directly tested whether this facilitation effect was the result of processing LSFs associated with a Navon figure's global structure. We thus replicated Experiment 3 with the exception that we contrast balanced the Navon stimuli in order to suppress their LSFs (see Supplementary Material). Similar to Experiment 3, observers preferred to classify hybrid images based on LSF content, irrespective of the Navon task completed; however, and in contrast to Experiment 3, LSF-based hybrid image categorization was slower following global than local Navon tasks.

## Experiment 1

The goal of Experiment 1 was to demonstrate the availability of both spatial frequencies in our hybrid images. We asked observers to complete a classification task in which they were required to indicate whether a cue word corresponded to a previously presented low-pass, high-pass, broadband, or hybrid image.

### Methods

#### Observers

Eight undergraduate students from Concordia University participated in this study in return for partial course credit. All observers self-reported normal or corrected-to-normal vision. The University Human Research Ethics Committee at Concordia University approved all experiments reported in this article and all observers provided written consent.

#### Stimuli and apparatus

Stimuli were presented on a 21-in. Viewsonic 225fb CRT monitor (1024 × 768 resolution; 100 Hz refresh rate) controlled by a Dell Precision T3400 core2 quad processor running Microsoft Windows 7. Experiment Builder (SR Research, Ottawa, Ontario) was used to display the stimuli and record the responses. All participants were seated 60 cm away from the screen, and their head position was controlled using a table-mounted chinrest.

Stimuli were 128 natural images (32 unique images of highways, cities, living rooms, and valleys, respectively) taken from the Sun image database (Xiao et al., 2010). All images were equalized for mean luminance and RMS contrast (as described in Appendix B of Loschky et al., 2007) and were presented on a gray background (*RBG* values = [128, 128, 128]; luminance of 52 cd/m^2^). These images were the same categories used by Schyns and Oliva (1994), who showed that their overall contrast was similar (i.e., the Fourier amplitude spectra of the images are highly correlated with one another). Images were broadband, low-pass (below 2 cycles deg^−1^ of visual angle), high-pass (above 6 cycles deg^−1^ of visual angle), or hybrid images. Hybrids were constructed by combining the low frequency components of one scene (e.g., a city) with the high frequency components of another scene (e.g., highway). Mathwork Matlab (ver. 2011b) was used to create the images. A total of 32,768 possible hybrid images were constructed by taking every possible combination of the four scene categories. All images were gray scaled, located in the center of the screen, and were 256 × 256 pixels.

#### Procedure

**Figure 1:**
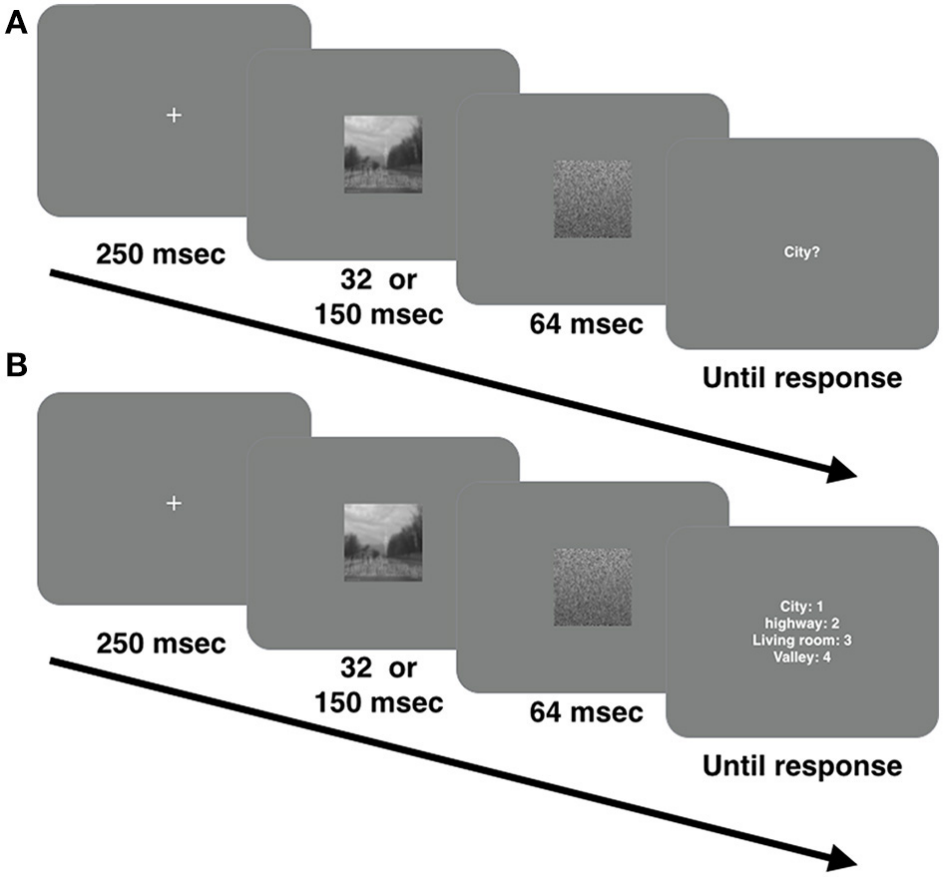
**(A)** Trial sequence in Experiment 1; **(B)** Trial sequence in Experiment 2.

A trial schematic is presented in Figure 1A. Each trial began with a fixation cross located in the center of the screen presented for 250 ms, followed by a single image presented for either 32, or 150 ms. A white noise mask (amplitude spectrum slope = 0; orientation magnitude = 0) immediately followed offset of the image and was presented for 64 ms. The image was a broadband, low-pass, high-pass, or a hybrid image. Immediately following offset of the mask, observers were presented with a display screen in which they were asked to indicate whether a probe word (e.g., highway, city, living room, or valley) corresponded to the category of the previously presented image. On 50% of trials, the cue word corresponded to the image category. Of those 50% of trials on which the image was a hybrid, the probe word matched the hybrid's LSF and HSF content 25% of the time, respectively. We instructed observers to press “1” on the keyboard number pad if they believed the probe word matched the previously presented image and the “2” key if they believed that it did not. The probe word was displayed in the center of the screen and stayed visible until a response was made. Trial-to-trial feedback was not provided.

Observers completed 16 blocks of 48 trials for a total of 768 trials. Image type and presentation duration varied from trial-to-trial within a block, and the order of images and presentation duration was chosen at random by the program. Observers completed 32 practice trials prior to beginning the experiment. The scene categories used during the practice trials were not used in the experimental trials (e.g., forest and barn scenes) and practice trials were not analyzed.

### Results

#### Sensitivity

**Figure 2:**
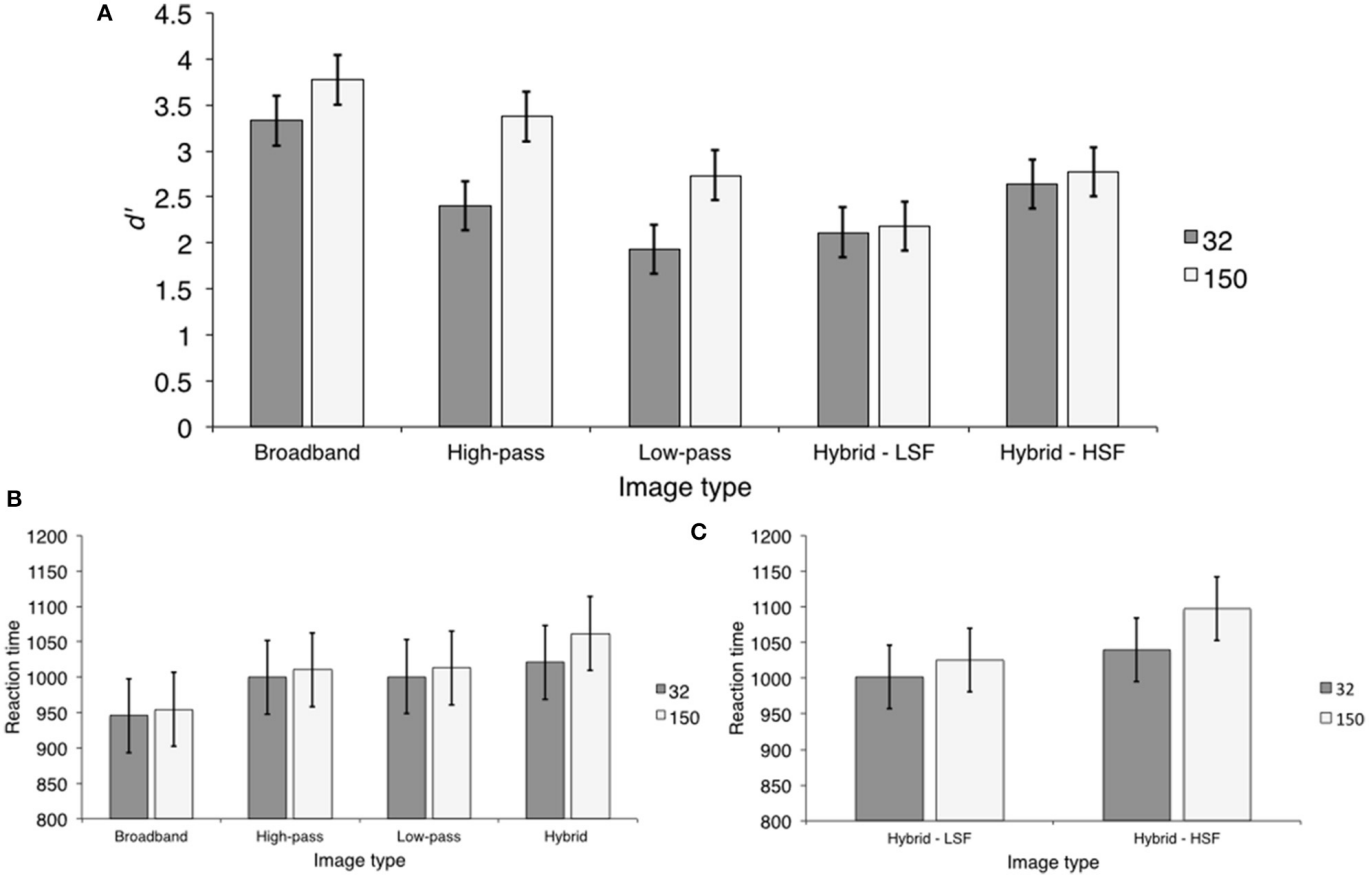
**The results of Experiment 1. (A)** *d*′ values for each image type at each presentation duration. The error bars represented here, and throughout the manuscript are the 95% within-subject confidence intervals described by Loftus and Masson (1994); **(B)** Mean scene categorization RTs for each image type at each presentation duration; **(C)** Mean RTs for hybrid-LSF and hybrid- HSF categorization at each presentation duration.

The sensitivity measure, *d*′ was calculated for each condition. Condition varied according to image type (broadband, low-pass, high-pass, and hybrid) and presentation duration (32 and 150 ms). Because hybrid images contained both low and HSF content, we further separated these trials into those on which the probe word matched the hybrid's low (Hybrid-LSF) and HSF content (Hybrid-HSF). As can be seen in Figure 2A, *d*′ values were high (*d*′ > 1.5) in all conditions, suggesting that observers were sensitive to all image types at both presentation durations. We entered *d*′ values into a 5 (image type) × 2 (presentation duration) repeated measures Analysis of Variance (ANOVA). There were significant main effects of image type, *F*_(4, 28)_ = 8.09, *p* < 0.001, η^2^ = 0.54, and presentation duration, *F*_(1, 7)_ = 34.47, *p* < 0.001, η^2^ = 0.83. The image type × presentation duration interaction was also significant, *F*_(4, 28)_ = 4.65, *p* < 0.001, η^2^ = 0.39.

Because Experiment 1 was designed to determine the availability of spatial frequencies in our hybrid images, we were particularly interested in comparisons between Hybrid-LSF and Hybrid-HSF trial types. However, before investigating the competing spatial scale information within hybrid images, we first compared performance between control images (low-pass, high-pass, and broadband), to ensure that our observers were sensitive to all spatial scales. We first computed the planned comparison comparing *d*′ values using a 3 (image type) × 2 (presentation duration) planned contrast. This contrast was not significant, suggesting that there was no statistical difference in spatial frequency processing as a function of presentation duration, *F*_(1, 7)_ = 1.38, *p* > 0.279, η^2^ < 0.01. We then compared sensitivity between control images using a series of contrast comparisons. Specifically, we computed contrasts comparing *d*′ values between broadband images and high-pass (Ψ_1_) and low-pass (Ψ_2_) filtered images, respectively. *d*′ statistics and the results of these contrasts are displayed in Table 1. Observers were more sensitive to broadband images (*M* = 3.55; *SD* = 0.45) than both high-pass (*M* = 2.88; *SD* = 0.62) and low-pass filtered images (*M* = 2.33; *SD* = 0.40). Observers were equally sensitive to low-pass and high-pass filtered images (Ψ_3_). The effect size measures in Experiment 1 paralleled the significance results. The largest effect sizes were between broadband images and low-pass (η^2^ = 0.76) and high-pass (η^2^ = 0.47) filtered images. The effect size between low-pass and high-pass filtered images was relatively smaller in comparison (η^2^ = 0.26).

Table 1***d prime* statistics for each image type at each presentation duration in Experiment 1**.

**Table:** 

**Trial type**	***d***′					
	**32 ms**			**150 ms**		
	***M***	***SD***	**95% CI**	***M***	***SD***	**95% CI**
Broadband	3.32	0.46	[2.94, 3.71]	3.77	0.58	[3.28, 4.26]
Low-pass	1.93	0.45	[1.56, 2.31]	2.74	0.59	[2.24, 3.23]
High-pass	2.40	0.75	[1.78, 3.02]	3.37	0.62	[2.85, 3.89]
Hybrid-LSF	2.11	0.26	[1.89, 2.32]	2.18	0.38	[1.86, 2.50]
Hybrid-HSF	2.64	0.52	[2.21, 3.07]	2.77	0.47	[2.37, 3.16]

**Table:** 

**CONTRASTS**							
**Contrast**	***df***	***F***	***p***	***M*_***D***_**	***SE*(*M*_***D***_)**	**95% CI(*M*_***D***_)**	**η^2^**
Ψ_1_	(1, 7)	6.18	<0.042	0.67	0.70	[0.09, 1.25]	0.47
Ψ_2_	(1, 7)	22.2	<0.002	1.22	0.24	[0.65, 1.79]	0.76
Ψ_3_	(1, 7)	2.50	>0.158	0.55	0.33	[−0.22, 1.33]	0.26
Ψ_4_	(1, 7)	13.7	<0.008	0.56	0.14	[0.22, 0.89]	0.66

d prime mean difference contrasts in Experiment 1.

Ψ_*1*_, d′ comparison between broadband images and high-pass filtered images.

Ψ_*2*_, d′ comparison between broadband images and low-pass filtered images.

Ψ_*3*_, d′ comparison between low-pass filtered and high-passed filtered images.

Ψ_*4*_, d′ comparison between Hybrid—LSF and Hybrid—HSF image types.

Following the control image type analysis, we computed the contrast comparing hybrid trial types (Hybrid—LSF and Hybrid—HSF) as a function of presentation duration. This was not statistically significant, *F*_(1, 7)_ = 0.137, *p* > 0.722, η^2^ < 0.01. We followed up this analysis by comparing sensitivity between hybrid trial types using a planned contrast, collapsing over presentation duration (Ψ_4_). Observers were more sensitive to hybrid-HSF image types (*M* = 2.71; *SD* = 0.49) than hybrid-LSF image types (*M* = 2.14; *SD* = 0.26). Furthermore, the associated effect size (η^2^ = 0.66) was similar to the effect sizes reported for the significant control contrasts, suggesting that observers were in fact more sensitive to HSFs than LSFs in the hybrid images.

#### Reaction time

We calculated mean reaction time (RT) measures for each trial type as a function of presentation duration. These means are displayed in Figure 2B. We entered these means into a 4 (image type) × 2 (presentation duration) repeated measures ANOVA. Unlike the calculation of *d*′ statistics, hybrid images were not separated further because target absent trials are the same between Hybrid—LSF and Hybrid—HSF trial types. The main effect of image type was significant, *F*_(3, 21)_ = 3.29, *p* < 0.04, η^2^ = 0.3 However, the main effect of presentation duration and the image type × presentation duration interaction were not: *F*_(1, 7)_ = 0.368, *p* > 0.563, η^2^ < 0.05 and *F*_(3, 21)_ = 0.009, >0.899, η^2^ < 0.001.

Similar to the sensitivity analysis, we were primarily interested in differences between Hybrid-HSF and Hybrid-LSF image types, but first report the results related to the control images. Specifically, we computed contrasts that paralleled the sensitivity comparisons. Reaction time statistics and mean difference contrasts are displayed in Table 2. Observers were faster to respond to broadband images (*M* = 950.04; *SD* = 58.18) than both high-pass (*M* = 1005.26; *SD* = 36.67) (Ψ_1_) and low-pass filtered images (*M* = 1007.03; *SD* = 48.75) (Ψ_2_). There was no RT difference between low-pass and high-pass filtered images (Ψ_3_). Consistent with the sensitivity analysis, the largest effect size was between broadband images and low-pass filtered images (η^2^ = 0.52) followed by the effect size for the difference between broadband images and high-pass filtered images (η^2^ = 0.38). The effect size between low-pass and high-pass filtered images was negligible (η^2^ < 0.01).

Table 2**Reaction time statistics for each image type at each presentation duration in Experiment 1**.

**Table:** 

**Trial type**	**Reaction time (ms)**					
	**32 ms**			**150 ms**		
	***M***	***SD***	**95% CI**	***M***	***SD***	**95% CI**
Broadband	945.56	78.42	[879.99, 1011.13]	954.51	76.04	[890.92, 1018.09]
Low-pass	1000.89	76.27	[937.11, 1064.67]	1013.17	77.22	[948.59, 1077.73]
High-pass	999.98	46.74	[960.89, 1039.07]	1010.54	52.44	[966.69, 1054.39]
Hybrid	1020.98	52.17	[977.35, 1064.61]	1061.45	41.90	[1026.42, 1096.49]
^*^Hybrid—LSF	1001.99	24.80	[981.25, 1022.73]	1025.64	14.56	[1013.46, 1037.82]
^*^Hybrid—HSF	1039.97	92.13	[962.93, 1117.00]	1097.27	75.02	[1034.54, 1160.01]

**Table:** 

**CONTRASTS**							
**Contrast**	***df***	***F***	***p***	***M*_***D***_**	***SE* (*M*_***D***_)**	**95% CI (*M*_***D***_)**	**η**^**2**^
Ψ_1_	(1, 7)	4.32	<0.050	55.22	22.84	[1.21, 109.24]	0.38
Ψ_2_	(1, 7)	7.58	<0.028	56.99	19.37	[11.18, 102.81]	0.52
Ψ_3_	(1, 7)	0.007	>0.937	1.76	20.11	[−45.81, 49.34]	<0.01

Reaction time mean difference contrasts in Experiment 1.

Ψ_1_, RT comparison between broadband images and high-pass filtered images.

Ψ_2_, RT comparison between broadband images and low-pass filtered images.

Ψ_3_, RT comparison between low-pass filtered and high-passed filtered images.

* Reaction time calculation is based on target present trials only.

Reaction times on target present trials were compared between Hybrid—LSF and Hybrid—HSF image types and are displayed in Figure 2C. We entered these means into a 2 (hybrid trial) × 2 (presentation duration) planned contrast. Consistent with the sensitivity analysis, this contrast was not significant, suggesting that RTs did not differ between hybrid image types as a function of presentation duration, *F*_(1, 7)_ = 0.617, *p* > 0.458, η^2^ < 0.08. We then compared RTs between hybrid—LSF and hybrid—HSF image types, collapsing over presentation duration. This contrast was significant, *F*_(1, 7)_ = 7.58, *p* < 0.028, η^2^ = 0.52. Observers were faster to respond to Hybrid—LSF image types (*M* = 1013.81; *SD* = 16.37) than Hybrid—HSF image types (*M* = 1068.62; *SD* = 41.90). This was a difference of approximately 54.81 ms (*SD* = 52.65; 95% CI [11.91, 97.71]). It is interesting to note that the associated effect size was similar to the effect size reported in the parallel sensitivity analysis (η^2^ = 0.66), suggesting that the effect of hybrid trial type is robust across dependent variables.

### Discussion

The critical result from Experiment 1 is that we corroborated Oliva and Schyns (1997) finding that both spatial scales are available to form the basis for hybrid image categorization. Observers in our study were sensitive to both sources of spatial frequency content and there was no significant interaction with presentation duration, although observers were overall more sensitive to HSFs than LSFs in the hybrid images. An interesting finding from Experiment 1 is that *d*′ values were overall high, which is suggestive of weak masking effects. The most likely explanation for this result is that we constructed our masks so that their amplitude spectrum slope (i.e., the slope that conveys amplitude and orientation information in an image) would have a value of 0. Hansen and Loschky (2013) found that white noise masks with this property are the least effective at masking natural scene stimuli, whereas white noise masks whose amplitude spectrum slope most closely resembled that of a natural scene (e.g., ~ alpha = 1; Hansen et al., 2008) are the most effective. This suggestion is consistent with previous studies that showed that the most effective mask for a particular spatial frequency is one whose amplitude spectrum information is most similar to the target stimuli (Stromeyer and Julesz, 1972; Losada and Mullen, 1995; Mullen and Losada, 1999).

## Experiment 2

Experiment 2 is an extension of Experiment 1. Whereas Experiment 1 assessed the availability of spatial scale information, Experiment 2 assessed diagnostic spatial scale preference between competing sources of LSF and HSF information. Thus, Experiment 2 is a replication of Experiment 1, with the exception that we assessed scene categorization using an all-alternative forced choice paradigm. We asked observers to choose which of all possible target categories corresponded to the previously presented hybrid image. Because a hybrid image's LSFs and HSFs convey information related to different categories, forcing observers to choose between all possible target categories indexes their preferred diagnostic spatial scale.

### Methods

#### Observers

Ten undergraduate students from Concordia University participated in this study in return for partial course credit. All observers self-reported normal or corrected-to-normal vision.

#### Stimuli, apparatus, and procedure

An example of a trial sequence in Experiment 2 is presented in Figure 1B. Stimuli, apparatus, and procedure were the same as in Experiment 1 with the following exception. Categorization performance was measured using a 4-alternative forced choice task. Immediately following offset of the mask, we presented observers with a list of 4 probe words with an associated number (city = 1, highway = 2, living room = 3, and valley = 4) listed vertically in the center of the screen. The task of the observer was to as quickly and as accurately as possible indicate the category of the previous image by pressing the corresponding key on the keyboard number pad.

### Results

#### Sensitivity

**Figure 3:**
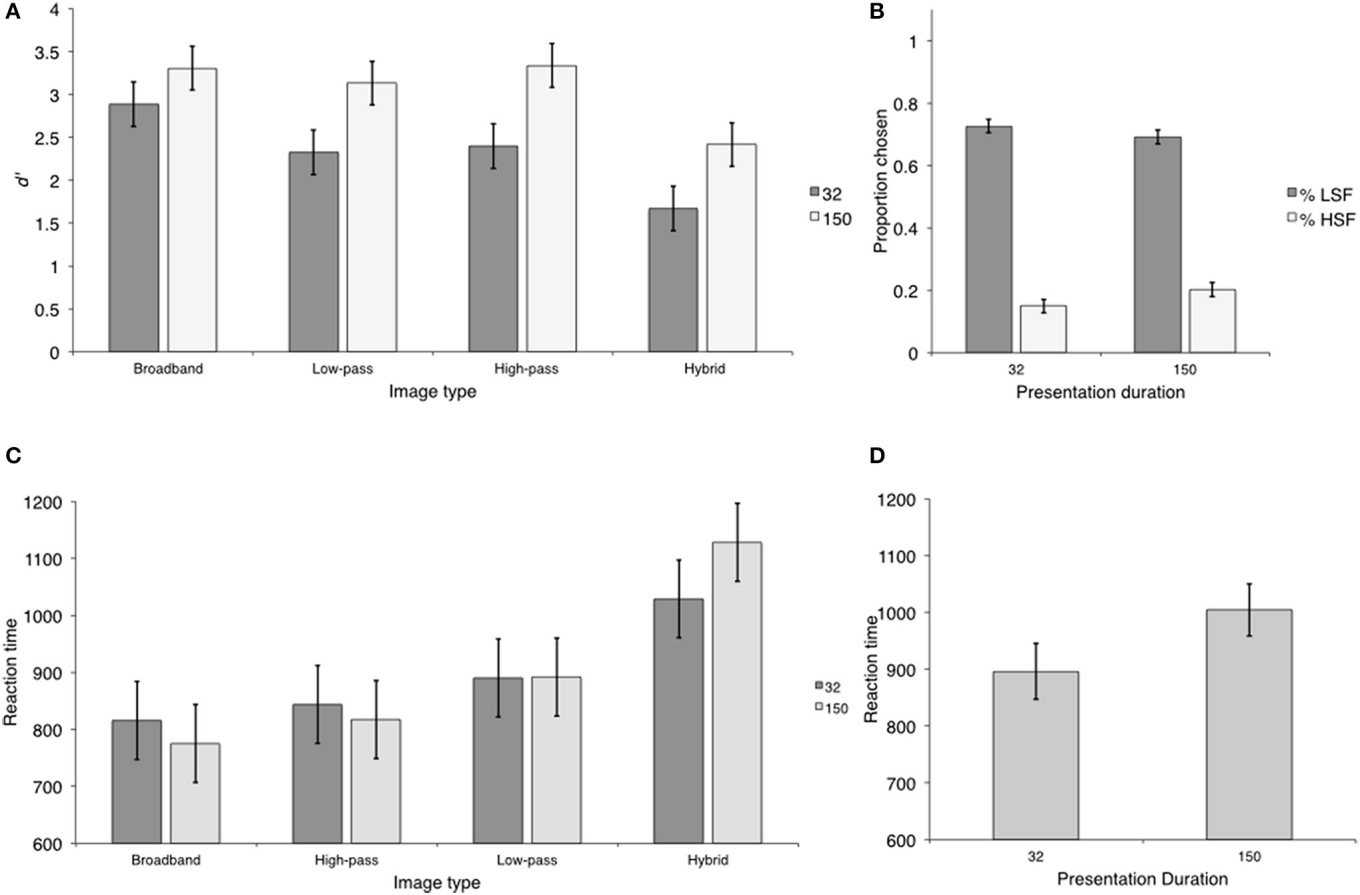
**The results of Experiment 2**. **(A)** *d*′ values for each image type at each presentation duration; **(B)** Percentage of low- and HSF-based hybrid categorization at each presentation duration; **(C)** Scene categorization RTs for each image type at each presentation duration; **(D)** Reaction times for LSF-based hybrid categorization at each presentation duration.

*d*′ was computed for each condition, by transforming proportion correct as described by Kingdom and Prins (2010), which assumes that there is no response bias. This transformation has specific consequences for the interpretation of results, which are discussed in the general discussion. These means are displayed in Figure 3A. Similar to Experiment 1, *d*′ values were above 1.5 in each condition, suggesting that observers were sensitive to all image types. We entered these means into a 2 (presentation duration) × 4 (image type) repeated measures ANOVA. There were significant main effects of image type, *F*_(3, 27)_ = 10.91, *p* < 0.001, η^2^ = 0.55, and presentation duration, *F*_(1, 9)_ = 56.83, *p* < 0.001, η^2^ = 0.86. The image type × presentation duration interaction was not significant, *F*_(3, 27)_ = 1.29, *p* > 0.299, η^2^ = 0.13. Observers were more sensitive at long (*M* = 3.05; *SD* = 0.29) than short (*M* = 2.32; *SD* = 0.13) durations, a difference of 0.73 (*SD* = 0.29; 95% CI [0.52, 0.94]). Although sensitivity was high in all conditions, the significant image type main effect appears to be driven by the fact that observers were less sensitive to hybrid images (*M* = 2.04; *SD* = 0.18) than the other image types (*M* = 2.89; *SD* = 0.21). This contrast (Ψ_1_) was statistically significant. Furthermore, the contrast comparing sensitivity between broadband images (*M* = 3.09; *SD* = 0.47) and low-pass and high-pass filtered images (*M* = 2.79; *SD* = 0.14) was not significant, corroborating our conclusion (Ψ_2_). Consistent with this conclusion, the effect size for Ψ_1_(η^2^ = 0.85) was higher than Ψ_2_(η^2^ = 0.31). *d*′ statistics and contrast analyses are displayed in Table 3.

Table 3***d prime* statistics for each trial type at each presentation duration in Experiment 2**.

**Table:** 

**Trial type**	***d*′**					
	**32 ms**			**150 ms**		
	***M***	***SD***	**95% CI**	***M***	***SD***	**95% CI**
Broadband	2.88	0.59	[2.50, 3.26]	3.31	0.38	[3.06 3.55]
Low-pass	2.33	0.59	[1.94, 2.71]	3.13	0.37	[2.89, 3.37]
High-pass	2.39	0.52	[2.06, 2.73]	3.34	0.65	[2.92, 3.75]
Hybrid	1.67	0.28	[1.49, 1.85]	2.41	0.17	[2.31 2.53]

**Table:** 

**CONTRASTS**							
**Contrast**	***df***	***F***	***P***	***M*_***D***_**	***SE*(*M*_***D***_)**	**95% CI (*M*_***D***_)**	**η**^**2**^
Ψ_1_	(1, 9)	48.46	<0.001	0.85	0.12	[0.39, 1.32]	0.85
Ψ_2_	(1, 9)	3.94	>0.078	0.29	0.14	[−0.28, 0.86]	0.31

d prime mean difference contrasts in Experiment 2.

Ψ_*1*_, d′ comparison between hybrid image types and the other image types.

Ψ_*2*_, d′ comparison between broadband images and low-pass and high pass filtered images.

In order to examine diagnostic spatial scale preference, we separated hybrid trials into those on which categorization was based on low and HSF content, respectively. As can be seen in Figure 3B, observers preferred to categorize hybrid images based on LSF content at both short and long presentation durations. HSF based hybrid categorization did not exceed chance at long durations (*M* = 0.20; *SD* = 0.07), *t*_(9)_ = 1.87, *p* > 0.095 and was worse than chance at short durations (*M* = 0.15; *SD* = 0.03), *t*_(9)_ = 8.64, *p* < 0.001. As a result, we concentrated our analysis on trials on which hybrid categorization was based on LSF content. LSF-based hybrid categorization did not statistically significantly differ between short (*M* = 0.73; *SD* = 0.08) and long (*M* = 0.69; *SD* = 0.09) durations, *t*_(9)_ = 1.78, *p* > 0.111, Cohen's *d* = 0.55, a difference of 0.04 (*SD* = 0.06; 95% CI [−0.01, 0.09]).

#### Reaction time

Reaction times were computed as described in the sensitivity analysis and are displayed in Figure 3C. We entered RTs into a 2 (presentation duration) × 4 (image type) repeated measures ANOVA. There was a significant main effect of image type, *F*_(3, 27)_ = 15.44, *p* < 0.001, η^2^ = 0.63. The main effect of presentation duration and the image type × presentation duration interaction were not significant, *F*_(1, 9)_ = 0.033, *p* > 0.860, η^2^ = 0.01 and *F*_(3, 27)_ = 1.77, *p* > 0.176, η^2^ = 0.16. Looking at Figure 3C, it is clear that observers were overall slower to respond to hybrid images (*M* = 1078.79; *SD* = 131.27) than any other image type (*M* = 838.87; *SD* = 43.76). This contrast was statistically significant (Ψ_1_). Furthermore, observers were faster to respond to broadband images (*M* = 795.27; *SD* = 41.91) than low-pass and high-pass filtered images (*M* = 860.66; *SD* = 49.58) (Ψ_2_). There was no significant difference in RTs between low-pass (*M* = 890.67; *SD* = 89.6) and high-pass filtered (*M* = 830.65; *SD* = 29.25) images (Ψ_3_). Similar to the previous experiments, effect size comparisons paralleled the significance results. The effect size associated with the non-significant difference between low-pass and high-pass filtered images was the smallest (η^2^ = 0.31), whereas the largest effect sizes were between broadband images and low-pass and high pass filtered images (η^2^ = 0.75) and between hybrid images and the other image types (η^2^ = 0.72). Reaction time statistics and contrast analyses are displayed in Table 4.

Table 4**Reaction time statistics for each trial type at each presentation duration in Experiment 2**.

**Table:** 

**Trial type**	**Reaction time (ms)**					
	**32 ms**			**150 ms**		
	***M***	***SD***	**95% CI**	***M***	***SD***	**95% CI**
Broadband	815.29	104.75	[740.37, 890.23]	775.25	101.48	[702.66, 847.84]
Low-pass	889.69	108.20	[812.44, 966.94]	891.66	115.17	[809.27, 974.04]
High-pass	844.03	93.96	[776.81, 911.24]	817.28	82.28	[758.42, 876.13]
Hybrid	1029.46	141.24	[928.42, 1130.49]	1128.08	148.12	[1022.13, 1234.03]
Hybrid—LSF	896.45	157.08	[739.36, 1053.54]	1004.67	144.88	[859.78, 1149.55]

**Table:** 

**CONTRASTS**							
**Contrast**	***df***	***F***	***p***	***M*_***D***_**	***SE* (*M*_***D***_)**	**95% CI (*M*_***D***_)**	**η**^**2**^
Ψ_1_	(1, 9)	23.64	<0.001	239.92	55.35	[114.69, 365.11]).	0.72
Ψ_2_	(1, 9)	27.65	<0.001	65.38	11.80	[38.70, 92.08]	0.75
Ψ_3_	(1, 9)	4.09	>0.074	60.02	28.17	[−3.07, 123.75]	0.31

Reaction time mean difference contrasts in Experiment 2.

Ψ_*1*_, RT comparison between hybrid images and the other image types.

Ψ_*2*_, RT comparison between broadband images and high-pass and low-pass filtered images.

Ψ_*3*_, RT comparison between high-pass and low pass filtered images.

As with the sensitivity analysis, our main goal was to index differences relating to hybrid images. However, because HSF-based categorization was no better (or worse) than chance, we restricted our hybrid RT analysis to trials on which hybrid categorization was based on LSF content (Figure 3D). LSF-based hybrid categorization was statistically significantly faster at short than long durations, *t*_(9)_ = 2.98, *p* < 0.016, Cohen's *d* = 0.94, a difference of 108.21 ms (*SD* = 109.12; 95% CI [30.15, 186.28]).

### Discussion

Experiment 2 showed that observers preferred to categorize hybrid images based on LSF content, at both short and long durations. However, an interesting finding is that observers were significantly slower at categorizing hybrid images compared to the other image types. The most likely explanation for this result is that although the probability of a correct answer was greatest for hybrid images (50 vs. 25%) their categorization nevertheless led to greater interference effects because they contained competing sources of diagnostic information. Together with the fact that HSF-based hybrid categorization did not exceed chance performance, and these results corroborate the finding that although observers process information at multiple spatial scales, they nevertheless use a single spatial scale as the basis for categorization (Oliva and Schyns, 1997). Along the same lines, observers in the current study were less sensitive to hybrid images than the other image types. Similar to above, the most parsimonious explanation for this result is that hybrid images differed from control images with respect to the probability of a correct answer. Because observers had a 50% chance at guessing the category of a hybrid image, this essentially reduced the 4-alternative forced choice task to a 2-alternative forced choice task. Thus, although accuracy was comparable between the different image types, sensitivity was nonetheless lower for hybrid images.

The critical finding from Experiment 2 is that observers overwhelmingly preferred to base hybrid image categorization on LSF content, despite the fact that both LSFs and HSFs were perceptually available (Experiment 1). The results of Experiments 1 and 2 thus serve as a baseline for Experiment 3 in which we examined whether we can influence diagnostic spatial selection by directing attention to either global, or local levels of hierarchical Navon figures.

## Experiment 3

Experiment 3 was a replication of Experiment 2 with the exception that we asked observers to complete global and local Navon tasks prior to classifying hybrid images. Similar to Experiments 1 and 2, we included control images in order to properly understand how attending locally and globally affected the processing of LSFs and HSFs. Because observers preferred to base hybrid categorization on LSF content, we predicted that LSF-based hybrid categorization would be facilitated following global Navon tasks; that is, LSF-based hybrid categorization would be faster following global Navon tasks than local Navon tasks. Also, because there was no interaction between presentation duration and categorization performance in Experiments 1 and 2, we simplified our design by presenting images at only 32 ms.

### Methods

#### Observers

Fourteen naïve undergraduate students from Concordia University participated in this study in return for partial course credit. All observers self-reported normal or corrected-to-normal vision.

#### Stimuli and apparatus

Stimuli and apparatus were the same as in Experiment 1 with the following exceptions.

#### Navon task

Stimuli used in the Navon task were white Navon letters (*RBG* values, [255, 255, 255]; luminance of 102 cd/m^2^) presented on a gray background (*RBG* values, [128, 128, 128]; luminance of 52 cd/m^2^). The display consisted of two Navon letters, one in the left and one in the right visual field, located 1° from a centrally located fixation cross. The global and local features of the Navon stimuli were either consistent (e.g., a large C comprised of copies of smaller Cs) or conflicting (e.g., a large T comprised of copies of smaller Cs). The letters used were C, E, H, and T, in all their global and local combinations. Each local letter subtended 0.7° × 0.7° of visual angle whereas the global letter subtended 5.7° × 4° of visual angle.

#### Scene categorization task

Stimuli in the scene categorization task were the same as in Experiment 1.

### Procedure

#### Navon task

Trials began with a fixation cross located at the center of the screen, presented for 250 ms, immediately followed by the presentation of the Navon letters, presented for 100 ms. The task of the participant was to indicate whether the local (local phase) or the global (global phase) configurations of the Navon letters matched. We instructed observers to press the “1” key on the keyboard number pad if they believed that the two Navon letters matched; we instructed observers to press the “2” key if they believed that they did not. Responses were speeded, and no trial-by-trial feedback was provided.

#### Scene categorization task

Each trial began with a fixation cross located in the center of the screen presented for 250 ms, followed by a single natural image presented for a display-to-mask SOA of 32 ms. A mask (the same white noise mask used in the previous experiments) followed image offset and was presented for 64 ms. The image was a broadband, low-pass, high-pass, or a hybrid image. Immediately following offset of the mask, observers were presented with a display screen in which they were asked to indicate the category of the image presented (e.g., city = 1, highway = 2, living room = 3, or valley = 4) by pressing the corresponding number of the category. The options were presented in the center of the screen and stayed visible until a response was made. Trial-to-trial feedback was not provided.

#### Design

**Figure 4:**
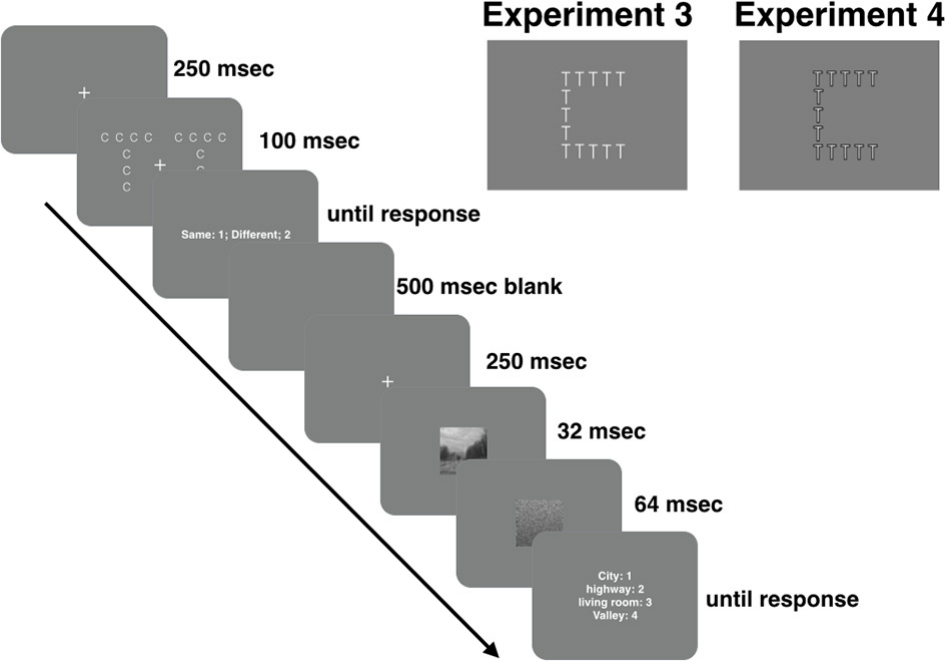
**Trial sequences in Experiments 3 and 4 and examples of regular and contrast balanced Navon stimuli**.

Observers completed two phases: a local phase and a global phase. In both phases, observers completed both the Navon task and the scene categorization task on each experimental trial (e.g., Navon task—scene categorization task—Navon task—scene categorization task; Martin et al., 2012). The only difference between the phases was whether observers were asked to indicate whether the local (local phase) or the global (global phase) configurations of the Navon letters matched. An example of a trial type is presented in Figure 4. There were an equal number of consistent and inconsistent Navon letters presented. The order in which observers completed the phases was counterbalanced across observers. There was a minimum of 30 min and a maximum of 60 min between phases. This was done to minimize any potential for interference between the different Navon tasks. Before the start of each phase, observers completed 30 practice trials in order to familiarize themselves with the task. Scene categories used during the practice trials were not used in the experimental trials (e.g., forests and barn scenes) and were not analyzed. Each phase consisted of 16 blocks of 48 trials for a grand total 768 trials.

To ensure that observers were primed to the appropriate attention level from the beginning of both the local and the global phases, observers first completed a respective block (48 trials) of either the global, or local Navon task. Similar to the practice trials, the main purpose of this priming block was to minimize any interference effects from the previous block. Trials in this phase were not analyzed.

### Results

#### Scene categorization results

The primary objective of Experiment 3 was to understand how attending to local and global levels of Navon figures affected the subsequent selection of diagnostic spatial scale information in subsequently presented hybrid images. However, and similar to Experiments 1 and 2, it was necessary that we first understood how attention to hierarchical level affected the processing of low and HSFs within our scenes. Accordingly, we first analyzed sensitivity and RT data between the control images.

**Figure 5:**
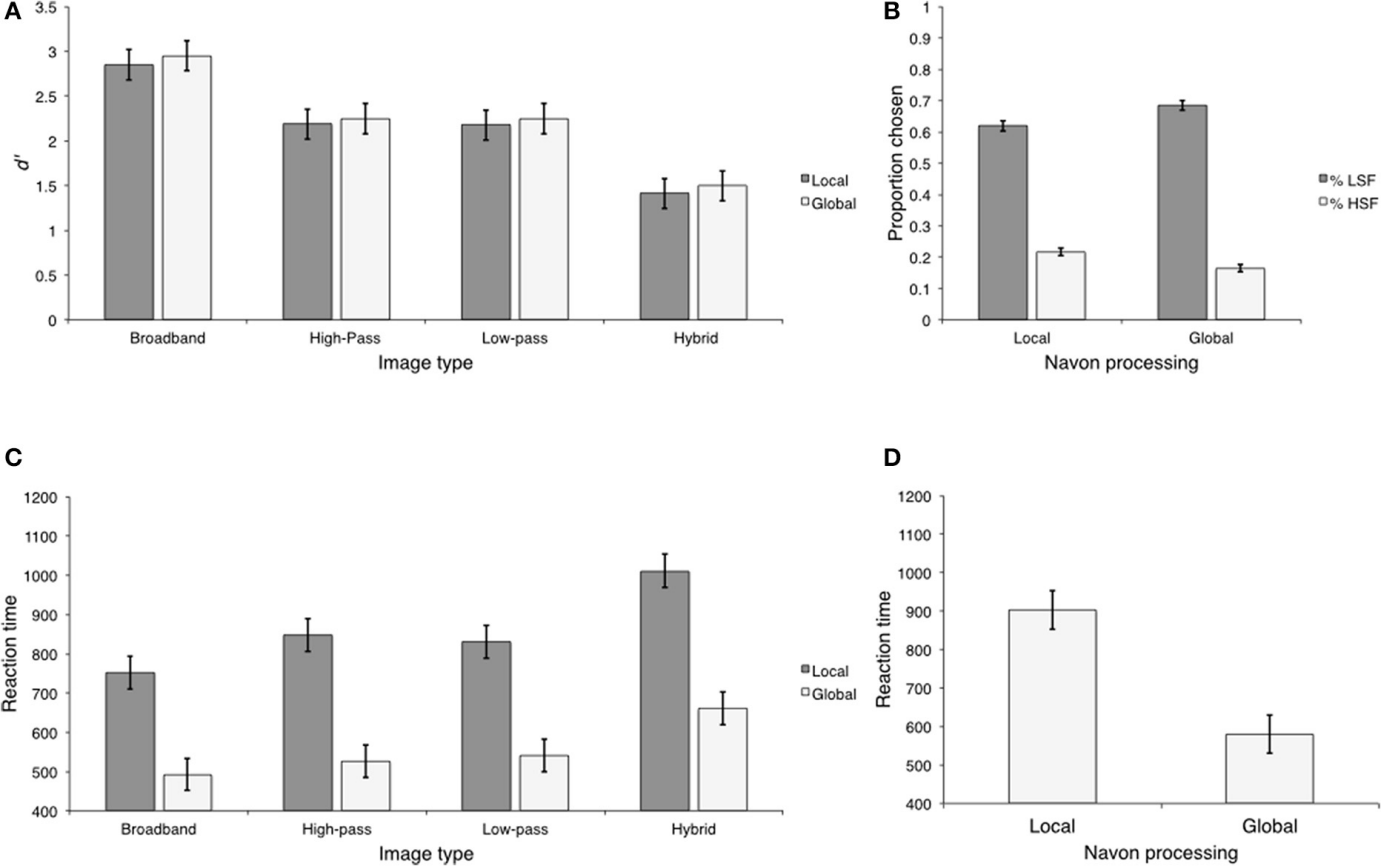
**The results of Experiment 3. (A)** Scene categorization accuracy for each image type for local and global Navon conditions; **(B)** Percentage of low- and HSF-based hybrid categorization for local and global Navon conditions; **(C)** Scene categorization RTs for each image type for local and global conditions; **(D)** Reaction times for LSF-based hybrid categorization for local and global conditions.

***Sensitivity.*** Mean *d*′ *values* were computed for each trial type. Trial type varied according to image type and Navon processing. These means are displayed in Figure 5A and were entered into a 2 (Navon) × 4 (image type) repeated measures ANOVA. There was a significant main effect of image type, *F*_(3, 39)_ = 40.15, *p* < 0.001, η^2^ = 0.75, but neither the main effect of Navon nor the image type × Navon interaction were significant, *F*_(1, 13)_ = 0.851, *p* > 0.373, η^2^ = 0.06 and *F*_(3, 39)_ = 0.027, *p* > 0.994, η^2^ = 0.02. Similar to Experiment 2, observers were less sensitive to hybrid images (*M* = 1.46; *SD* = 0.27) than the other image types (*M* = 2.44; *SD* = 0.11) (Ψ_1_). Furthermore, observers were more sensitive to broadband images (*M* = 2.90; *SD* = 0.42) than low-pass and high-pass filtered images (*M* = 2.21; *SD* = 0.22) (Ψ_2_). There was no difference in sensitivity between low-pass and high-pass filtered images (Ψ_3_). As in Experiment 2, the effect sizes associated with Ψ_1_(η^2^ = 0.88) and Ψ_2_(η^2^ = 0.73) were similar, replicating the result that observers were most sensitive to broadband images and least sensitive to hybrid images. The effect size between low-pass and high-pass filtered images was relatively smaller (η^2^ = 0.07). *d*′ statistics and contrast analyses are displayed in Table 5.

Table 5***d prime* statistics for each trial type in local and global conditions in Experiment 3**.

**Table:** 

**Trial type**	***d*′**					
	**32 ms**			**150 ms**		
	***M***	***SD***	**95% CI**	***M***	***SD***	**95% CI**
Broadband	2.84	0.52	[2.56, 3.14]	2.95	0.56	[2.63, 3.27]
Low-pass	2.18	0.37	[1.97, 2.38]	2.25	0.22	[2.12, 2.37]
High-pass	2.19	0.36	[1.98, 2.39]	2.25	0.21	[2.11, 2.36]
Hybrid	1.41	0.29	[1.24, 1.58]	1.49	0.28	[1.34, 1.66]

**Table:** 

**CONTRASTS**							
**Contrast**	***df***	***F***	***P***	***M*_***D***_**	***SE(M*_***D***_)**	**95% CI (*M*_***D***_)**	**η**^**2**^
Ψ_1_	(1, 13)	94.06	<0.001	0.98	0.09	[0.62, 1.36]	0.88
Ψ_2_	(1, 13)	17.27	<0.001	0.69	0.16	[0.09, 1.28]	0.73
Ψ_3_	(1, 13)	1.01	>0.336	<0.01	0.01	[−0.01, 0.03]	0.07

d prime mean difference contrasts in Experiment 3.

Ψ_1_, d′ comparison between hybrid images and the other image types.

Ψ_2_, d′ comparison between broadband images and high-pass and low-pass filtered images.

Ψ_3_, d′ comparison between low-pass and high-pass filtered images.

The proportion of low- and HSF-based hybrid categorization is displayed in Figure 5B. As in Experiment 2, observers preferred to classify hybrid images based on LSF content in both local and global conditions. Furthermore, HSF-based hybrid categorization was no better than chance in the local condition (*M* = 0.22; *SD* = 0.02), *t*_(13)_ = 1.61, *p* > 0.133, Cohen's *d* = 0.14, and worse than chance in the global condition (*M* = 0.17; *SD* = 0.04), *t*_(13)_ = 3.83, *p* < 0.001, Cohen's *d* = 0.51. Thus, we restricted our analysis to trials on which hybrid categorization was based on LSF content. LSF-based hybrid categorization was higher following global (*M* = 0.69; *SD* = 0.11) than local Navon tasks (*M* = 0.62; *SD* = 0.11), *t*_(13)_ = 4.29, *p* < 0.001, Cohen's *d* = 1.14, a difference of 0.07 (*SD* = 0.06; 95% CI [0.04, 0.10]).

***Reaction time.*** Mean RTs were computed as in the sensitivity analysis and are displayed in Figure 5C. We entered these means into a 2 (Navon) × 4 (image type) repeated measures ANOVA. There were significant main effects of image type, *F*_(3, 39)_ = 16.15, *p* < 0.001, η^2^ = 0.55, and Navon, *F*_(1, 13)_ = 98.55, *p* < 0.001, η^2^ = 0.88. The Navon × image type interaction was not significant, *F*_(3, 39)_ = 2.07, *p* > 0.121, η^2^ = 0.14. Reaction times were overall faster following global (*M* = 555.41; *SD* = 59.94) than local Navon tasks (*M* = 860.22; *SD* = 94.38), a difference of 304.81 ms (*SD* = 110.70; 95% CI [211.31, 398.31]).

As in Experiment 2, the significant image type main effect appears to be due to the fact that RTs were slower in response to hybrid images. The contrast comparing RTs between hybrid image types (*M* = 835.87; *SD* = 67.77) and the other image types (*M* = 665.14; *SD* = 33.88) was significant (Ψ_1_). Furthermore, RTs were faster for broadband images (*M* = 622.15; *SD* = 78.86) than low and high-pass filtered images (*M* = 686.63; *SD* = 34.33), corroborating the result from Experiment 2 (Ψ_2_). There was no significant difference between low-pass (*M* = 685.89; *SD* = 50.01) and high-pass filtered images (*M* = 687.37; *SD* = 82.49) (Ψ_3_). As in the previous experiments, the associated effect sizes mirrored the statistical significance results. The largest effect sizes were for Ψ_1_(η^2^ = 0.81) and Ψ_2_(η^2^ = 0.35), corroborating the finding that observers were overall fastest to respond to broadband images and slowest to respond to hybrid images. Furthermore, and similar to the sensitivity analysis, the effect size for the comparison between low-pass and high pass filtered images was small (η^2^ < 0.01), corroborating the finding that there were no meaningful differences between these image types. Reaction time statistics and contrast analyses are displayed in Table 6.

Table 6**Reaction time statistics for each image type in local and global conditions in Experiment 3**.

**Table:** 

**Trial type**	**Reaction time (ms)**					
	**Local**			**Global**		
	***M***	***SD***	**95% CI**	***M***	***SD***	**95% CI**
Broadband	751.40	80.49	[704.93, 797.86]	492.89	110.74	[428.96, 556.83]
Low-pass	830.50	78.04	[785.45, 875.59]	541.29	59.45	[506.96, 575.61]
High-pass	847.93	136.12	[769.34, 926.51]	526.81	72.29	[485.08, 568.54]
Hybrid	1011.06	150.13	[924.39, 1097.74]	660.65	70.20	[620.12, 701.18]
Hybrid—LSF	902.86	86.55	[852.89, 952.82]	580.53	86.55	[530.56, 630.49]

**Table:** 

**CONTRASTS**							
**Contrast**	***df***	***F***	***p***	***M*_***D***_**	***SE* (*M*_***D***_)**	**95% CI (*M*_***D***_)**	**η**^**2**^
Ψ_1_	(1, 13)	55.61	<0.001	170.71	101.65	[112.04, 282.79]	0.81
Ψ_2_	(1, 13)	7.02	<0.021	64.48	23.44	[13.83, 115.13]	0.35
Ψ_3_	(1, 13)	0.002	>0.965	1.47	117.89	[−66.58, 69.53]	<0.01

Reaction time mean difference contrasts in Experiment 3.

Ψ_*1*_, RT comparison between hybrid images and the other trial types.

Ψ_*2*_, RT comparison between broadband images and high-pass and low-pass filtered images.

Ψ_*3*_, RT comparison between high-pass and low-pass filtered images.

As in Experiment 2, we compared RTs between trials on which hybrids were classified according their LSF content (Figure 5D). LSF-based hybrid categorization was faster following global compared to local Navon tasks, *t*_(13)_ = 6.71, *p* < 0.001, Cohen's *d* = 1.79, a difference of 322.32 ms (*SD* = 173.12; 95% CI [222.38, 422.26]).

### Navon results

#### Accuracy

Mean accuracy was computed for the trial types described above. Overall, accuracy was above 90% in all conditions. We entered mean accuracy into a 2 (Navon) × 4 (image type) repeated measures ANOVA. The main effects of image type and Navon were not statistically significant, *F*_(3, 39)_ = 2.25, *p* > 0.098, η^2^ = 0.15, and *F*_(1, 13)_ = 0.126, *p* > 0.728, η^2^ = 0.01. Furthermore, the Navon × image type interaction was also not significant, *F*_(3, 39)_ = 1.19, *p* > 0.326, η^2^ = 0.09.

#### Reaction time

**Figure 6:**
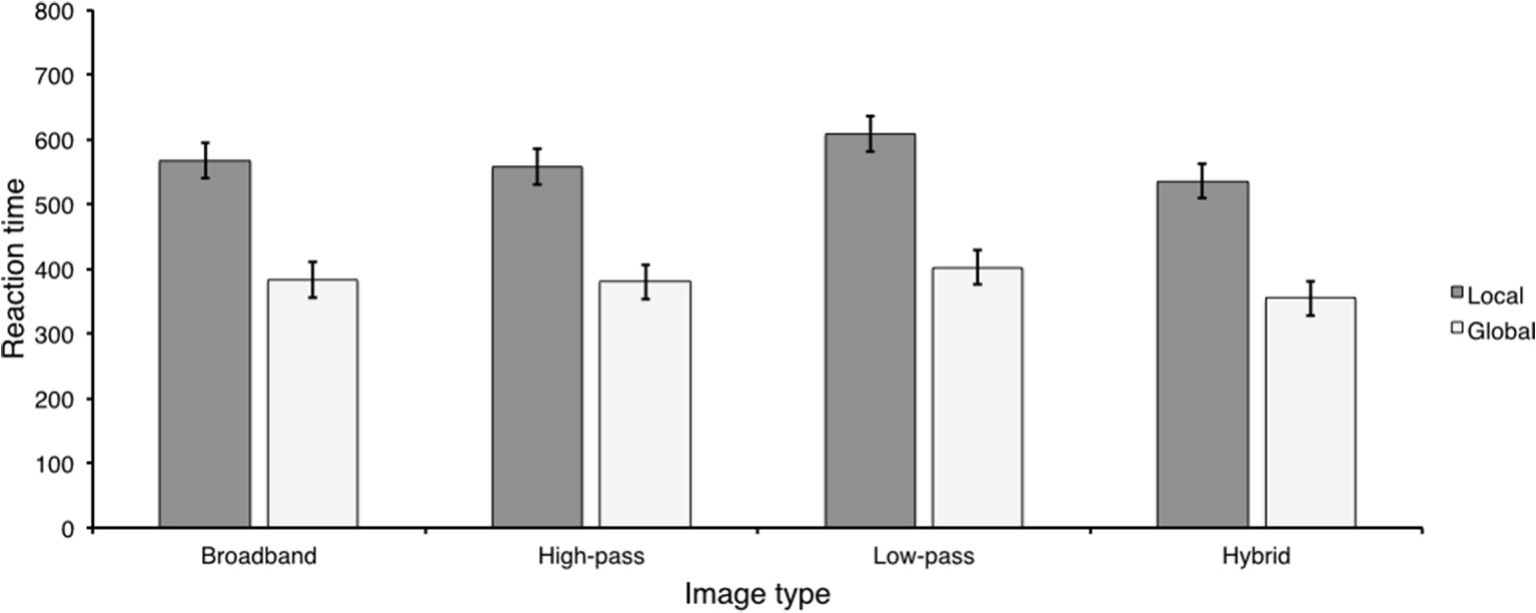
**Mean Navon RTs for each image type in Experiment 3**.

Mean RTs were computed as in the accuracy analysis and are displayed in Figure 6. We entered these means into a 2 (Navon) × 4 (image type) repeated measures ANOVA. There was a significant main effect of image type, *F*_(3, 39)_ = 6.88, *p* < 0.001, η^2^ = 0.35, and Navon, *F*_(1, 13)_ = 56.28, *p* < 0.001, η^2^ = 0.81. The Navon × image type interaction was not significant, *F*_(3, 39)_ = 0.449, *p* > 0.719, η^2^ = 0.03. Overall, global Navon tasks (*M* = 379.74; *SD* = 140.10) were completed faster than local Navon tasks (*M* = 567.07; *SD* = 146.40), This difference was approximately 187.34 ms (*SD* = 90.03; 95% CI [111.29, 263.38]) and corroborated the robust finding of the global precedence effect (Navon, 1977). The main effect of image type appears to be driven by the fact that Navon RTs were overall slowest when completed in conjunction with low-pass filtered images. A significant contrast comparing Navon RTs between low-pass filtered image trials (*M* = 505.42; *SD* = 148.53) and the other image trials (*M* = 562.73, *SD* = 132.82) confirmed this interpretation, *F*_(1, 13)_ = 59.14, *p* < 0.001, η^2^ = 0.42. This difference was approximately 57.31 ms (*SD* = 25.99; 95% CI [41.52, 73.09]). The contrast comparing Navon RTs between broadband image trials (*M* = 445.04, *SD* = 130.62) and the combined mean of high-pass filtered image trials and hybrid image trials (*M* = 471.58; *SD* = 136.61) was not significant, corroborating this conclusion, *F*_(1, 13)_ = 4.17, *p* > 0.071, η^2^ = 0.24. This difference was approximately 26.54 ms (*SD* = 46.85; 95% CI [−0.05, 53.58]).

### Discussion

Global Navon tasks were completed faster than local Navon tasks in Experiment 3, corroborating the global precedence effect (Navon, 1977). A critical result from Experiment 3 is that when given the choice between differing sources of diagnostic information, observers preferred to categorize hybrids based on LSF content, irrespective of the Navon task completed. Similar to Experiment 2, HSF-based categorization was no better than chance. Consistent with our hypothesis, LSF-based hybrid image categorization was statistically significantly faster following global than local Navon tasks. These results can be interpreted to suggest that we replicated Flevaris et al.'s (2011) finding that attending globally facilitated the selection LSFs in our hybrid images. However, this interpretation is inconsistent with the finding that both low-pass and high-pass filtered images were categorized faster following global Navon tasks. If attending locally and globally facilitated HSF and LSF processing, respectively, then high-pass filtered images should have been identified faster following local Navon tasks. According to Flevaris and colleagues, however, the selection of spatial frequencies is relative. Thus, although low-pass and high-pass filtered images have had HSFs and LSFs removed there are nevertheless still LSFs and HSFs within both image types. Thus, it is possible that the processing of LSFs associated with global Navon tasks facilitated the relatively LSFs in both low-pass and high-pass filtered images. This explanation seems likely given that observers preferred to categorize hybrid images based on LSF content. A prediction of this account is that removing a Navon's LSFs should eliminate the benefit associated with categorization following global Navon tasks. In Experiment 4, we directly tested this hypothesis by replicating Experiment 3 with the exception that we used contrast balanced Navon stimuli.

## Experiment 4

Experiment 4 was a replication of Experiment 3 with the exception that Navon stimuli were contrast balanced to suppress LSF information in order to encourage observers to use HSFs to accomplish both local and global Navon tasks. We confirmed that LSFs were reduced in the stimuli used in Experiment 4, by calculating the log-power spectra and rotationally averaged log amplitude spectra of both the contrast balanced and original Navon stimuli. These analyses are described in the Appendix. We predicted that forcing observers to use HSFs to complete Navon tasks, irrespective of attended level, would eliminate the global advantage associated with scene categorization observed in Experiment 3.

### Methods

#### Observers

Fifteen naïve undergraduate students from Concordia University participated in this study in return for partial course credit. All observers self-reported normal or corrected-to-normal vision.

#### Stimuli, apparatus, and procedure

Stimuli, apparatus, and procedure were the same as in Experiment 3, expect that Navon stimuli were contrast balanced, such that darker lines surrounded the white lines of the local letters.

### Results

#### Scene categorization results

**Figure 7:**
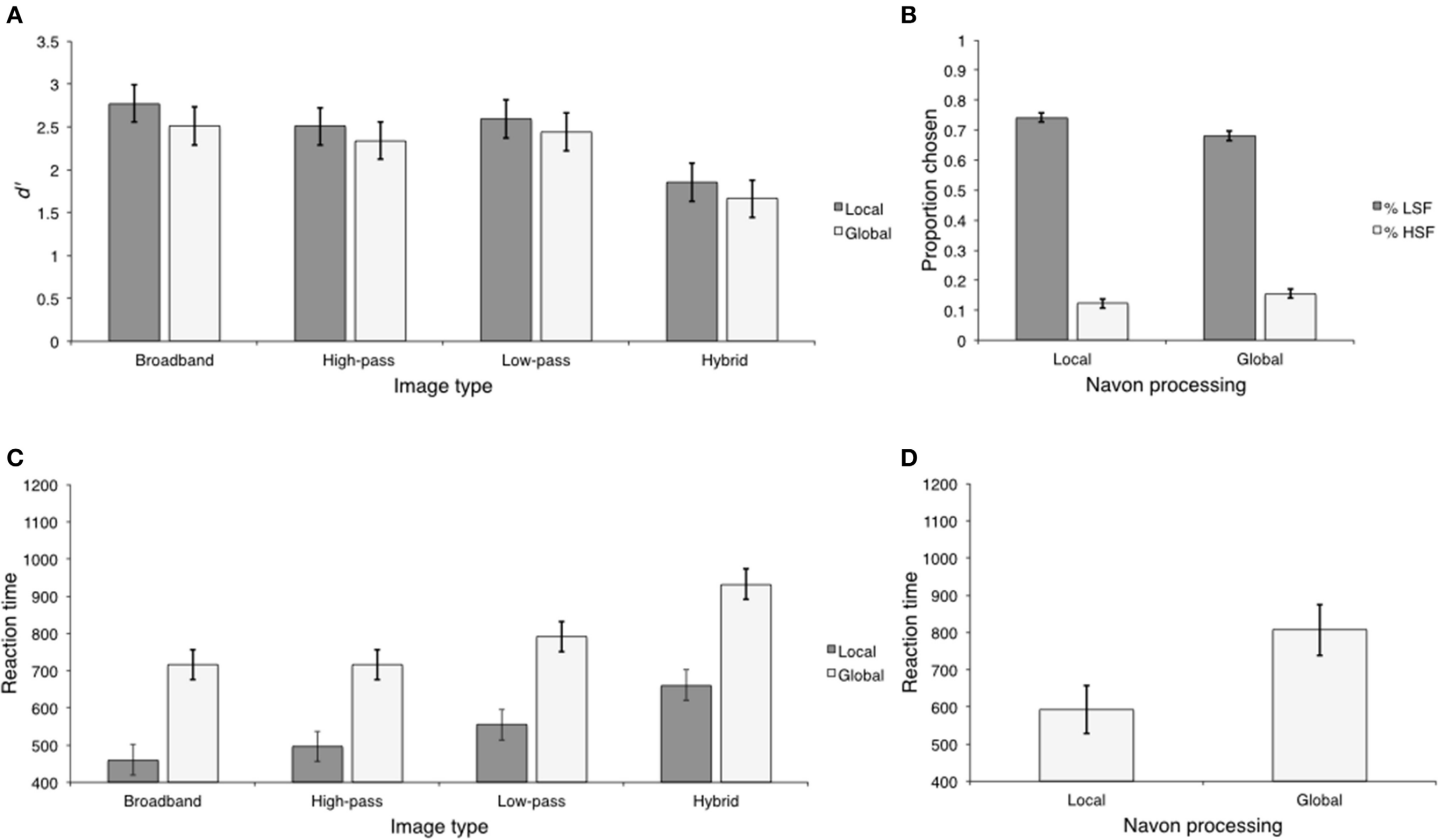
**The results of Experiment 4**. **(A)** *d*′ values for each image type for local and global Navon conditions; **(B)** Percentage of low- and HSF-based hybrid categorization for local and global Navon conditions; **(C)** Scene categorization RTs for local and global Navon conditions **(D)** Reaction times for LSF-based hybrid categorization for local and global conditions.

***Sensitivity.*** *d*′ values were computed as in Experiment 3 and are displayed in Figure 7A. Overall sensitivity was high, replicating performance in the previous experiments. We entered *d*′ means into a 2 (Navon) × 4 (image type) repeated measures ANOVA. There was a significant main effect of image type, *F*_(3, 42)_ = 15.41, *p* < 0.001, η^2^ = 0.52, but neither the main effect of Navon nor the image type × Navon interaction was significant, *F*_(1, 14)_ = 1.14, *p* > 0.304, η^2^ = 0.08 and *F*_(3, 42)_ = 0.196, *p* > 0.898, η^2^ = 0.14. Similar to previous experiments, observers were less sensitive to hybrid images (*M* = 1.75; *SD* = 0.29) than the other image types (*M* = 2.53; *SD* = 0.14) (Ψ_1_). Furthermore, there was no difference in sensitivity between broadband images (*M* = 2.64; *SD* = 0.13) and low-pass and high-pass filtered images (*M* = 2.47; *SD* = 0.11) (Ψ_2_). Furthermore, the effect size measures mirrored the statistical significance results. The effect size for Ψ_1_(η^2^ = 0.79) was larger than the effect size for Ψ_2_(η^2^ = 0.09), corroborating the finding that observers were less sensitive to hybrid images and equally sensitive to all other image types in Experiment 4. *d*′ statistics and contrast analyses are displayed in Table 7.

Table 7***d prime* statistics for each image type in local and global conditions in Experiment 4**.

**Table:** 

**Trial type**	***d*′**					
	**Local**			**Global**		
	***M***	***SD***	**95% CI**	***M***	***SD***	**95% CI**
Broadband	2.77	0.79	[2.41, 3.13]	2.51	0.62	[2.23, 2.79]
Low-pass	2.59	0.51	[2.36, 2.82]	2.44	0.19	[2.35, 2.53]
High-pass	2.50	0.44	[2.31, 2.71]	2.34	0.46	[2.13, 2.55]
Hybrid	1.85	0.49	[1.62, 2.08]	1.66	0.39	[1.49, 1.84]

**Table:** 

**CONTRASTS**							
**Contrast**	***df***	***F***	***P***	***M*_***D***_**	***SE(M*_***D***_)**	**95% CI *(M*_***D***_)**	**η**^**2**^
Ψ_1_	(1, 14)	50.35	<0.001	0.77	0.11	[0.58, 0.95]	0.79
Ψ_2_	(1, 14)	1.13	>0.269	0.17	0.14	[−0.08, 0.42]	0.09

d prime mean difference contrasts in Experiment 4.

Ψ_*1*_, d′ comparison between hybrid images and the other image types.

Ψ_*2*_, d′ comparison between broadband images and high-pass and low pass filtered images.

Similar to the previous experiments, observers preferred to categorize hybrid images based on LSF content in both local and global conditions (Figure 7B). Furthermore, HSF-based hybrid categorization was worse than chance following both local and global Navon tasks, *t*_(14)_ = 12.43, *p* < 0.001; and *t*_(14)_ = 6.14, *p* < 0.001. In contrast to experiment 3, LSF-based hybrid categorization was higher following local than global Navon tasks, *t*_(13)_ = 3.93, *p* < 0.001, Cohen's *d* = 1.07, a difference of 0.06 (*SD* = 0.06, 95% CI = [0.01, 0.11]). It is interesting to note that the effect size was consistent with the value reported in Experiment 3 (Cohen's *d* = 1.14), but is in the opposite direction, suggesting a complete reversal of the effect.

***Reaction time.*** Mean reaction time was computed as in Experiment 3 and is displayed in Figure 7C. Mean RTs were entered into a 2 (Navon) × 4 (image type) repeated measures ANONA. There were significant main effects of image type and Navon, *F*_(3, 42)_ = 23.56, *p* < 0.001, η^2^ = 0.63 and *F*_(1, 14)_ = 20.99, *p* < 0.001, η^2^ = 0.60. The Navon × image type interaction was not statistically significant, *F*_(3, 42)_ = 0.942, *p* > 0.429, η^2^ = 0.06. In contrast to Experiment 3, RTs were overall faster following local (*M* = 543.14; *SD* = 100.37) than global (*M* = 788.98; *SD* = 100.08) Navon tasks, a difference of 245.84 ms (*SD* = 200.74; 95% CI [104.89, 386.77]). As in the previous experiments, observers were slower to respond to hybrid image types (*M* = 796.71; *SD* = 86.81) than the other image types (*M* = 622.52; *SD* = 28.93) (Ψ_1_). Observers were also faster to respond to broadband images (*M* = 588.69; *SD* = 56.34) than low-pass and high-pass filtered images (*M* = 639.42; *SD* = 34.67) (Ψ_2_). In contrast to previous experiments, observers were faster to respond to high-pass filtered images (*M* = 606.22; *SD* = 49.23) than low-pass filtered images (*M* = 672.63; *SD* = 52.12) (Ψ_3_).

The largest effect size in Experiment 4 was for Ψ_1_(η^2^ = 0.80), corroborating previous experiments that observers were slowest to respond to hybrid images. Furthermore, the effect size for Ψ_2_(η^2^ = 0.36) was similar to the previous experiments, corroborating the finding that observers were fastest to respond to broadband images. However an interesting finding is that the effect size for Ψ_3_(η^2^ = 0.59) was relatively higher than those reported in previous experiments, suggesting that whereas there was no difference in RTs between low-pass and high-pass filtered images in Experiments 1–3, observers took longer to respond to high-pass filtered images than low-pass filtered images in Experiment 4. Reaction time statistics and contrast analyses are displayed in Table 8.

Table 8**Reaction time statistics for each image type in local and global conditions in Experiment 4**.

**Table:** 

**Trial type**	**Reaction time (ms)**					
	**Local**			**Global**		
	***M***	***SD***	**95% CI**	***M***	***SD***	**95% CI**
Broadband	460.32	111.15	[384.66, 535.98]	717.07	110.99	[641.78, 792.36]
Low-pass	554.38	116.30	[475.49, 633.27]	790.88	115.28	[712.68, 869.07]
High-pass	496.61	87.15	[437.50, 555.72]	715.83	121.62	[633.33, 798.33]
Hybrid—LSF	592.35	125.35	[528.09, 656.39]	821.97	115.91	[758.83, 885.13]
Hybrid	661.26	160.27	[552.55, 769.98]	932.15	155.22	[826.85, 1037.43]

**Table:** 

**CONTRASTS**							
**Contrast**	***df***	***F***	***p***	***M*_***D***_**	***SE* (*M*_***D***_)**	**95% CI (*M*_***D***_)**	**η**^**2**^
Ψ_1_	(1, 14)	54.99	<0.001	174.19	36.60	[95.68, 252.69]	0.80
Ψ_2_	(1, 14)	7.83	<0.014	50.73	21.45	[4.71, 96.74]	0.36
Ψ_3_	(1, 14)	20.64	<0.001	66.41	23.39	[16.23, 116.59]	0.59

Reaction time mean difference contrasts in Experiment 4.

Ψ_*1*_, Reaction time comparison between hybrid images and the other image types.

Ψ_*2*_, Reaction time comparison between broadband images and low-pass and high-pass filtered images.

Ψ_*3*_, Reaction time comparison between low-pass and high-pass filtered images.

As in previous experiments, we compared LSF-based hybrid categorization RTs between local and global conditions (Figure 7D). In contrast to Experiment 3, LSF-based hybrid categorization was statistically significantly faster following local than global Navon tasks, *t*_(14)_ = 3.21, *p* < 0.006, Cohen's *d* = 0.91, a difference of 229.62 (*SD* = 250.69, 95% CI [84.90, 374.34]). Furthermore, the associated effect size was relatively smaller than in Experiment 3 (Cohen's *d* = 1.79), suggesting that although the effect in Experiment 4 reversed direction, its magnitude is smaller.

### Navon results

#### Accuracy

Mean accuracy was computed as in Experiment 3 and replicated the overall high accuracy observed in the previous experiment (>90%). We compared accuracy by computing a 2 (Navon) × 4 (image type) repeated measures ANOVA. The main effects of Navon and image type were not significant, *F*_(1, 14)_ = 0.736, *p* > 0.405, η^2^ = 0.05 and *F*_(3, 42)_ = 0.628, *p* > 0.601, η^2^ = 0.04. The Navon × image type interaction was also not significant, *F*_(3, 42)_ = 0.301, *p* > 0.825, η^2^ = 0.02.

#### Reaction time

**Figure 8:**
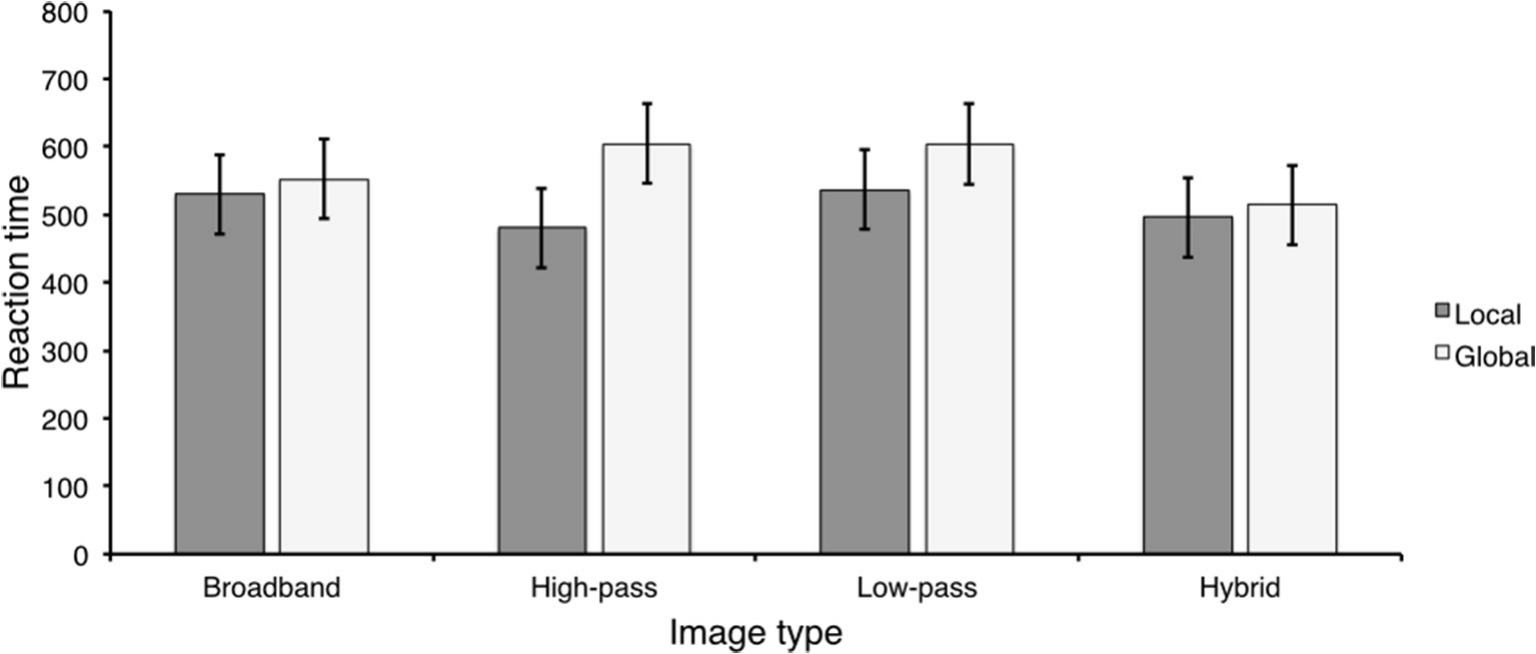
**Mean Navon RTs for each image type in Experiment 4**.

Mean RTs were computed as in the accuracy analysis and are displayed in Figure 8. We entered group mean RTs into a 2 (Navon) × 4 (image type) repeated measures ANONA. The main effects of Navon and image type were not significant, *F*_(3, 42)_ = 1.15, *p* > 0.226, η^2^ = 0.1 and *F*_(1, 14)_ = 0.924, *p* > 0.353, η^2^ = 0.06. Further, the Navon × image type interaction was not significant, *F*_(3, 42)_ = 1.52, *p* > 0.223, η^2^ = 0.10.

### Discussion

Experiment 4 was a replication of Experiment 3 with the exception that Navon stimuli were contrast balanced. There was no RT difference between Navon tasks, corroborating the previous finding that contrast balancing Navon stimuli eliminates the global precedence effect (Lamb and Yund, 1993). This forced observers to complete both local and global Navon tasks using HSFs. This afforded the opportunity to determine whether the observed global advantage in Experiment 3 was due to LSF processing associated with global Navon tasks.

An interesting result in Experiment 4 is that observers were faster to respond to high-pass filtered images than low-pass filtered images. One explanation for this result is that suppressing LSFs in Navon stimuli forced observers to complete Navon tasks using HSFs, which in turn, primed the selection of HSFs in high-pass filtered images. As in Experiment 3, observers preferred to categorize hybrid images based on LSF content, following both global and local Navon tasks. However, and in contrast, LSF-based hybrid categorization was slower following global than local Navon tasks. Thus, our prediction that contrast balancing Navon stimuli would eliminate the observed advantage for LSF-based hybrid image categorization following global Navon tasks in Experiment 3 was supported, although we did not predict a complete reversal of the effect. Furthermore, control images were all classified faster following local Navon tasks, suggesting that the global scene categorization advantage in Experiment 3 was due, in part, to the LSFs present in Navon stimuli.

## General discussion

The four experiments reported in this article investigated how attending to local and global levels of hierarchical Navon figures affected the selection of the diagnostic spatial scale used for scene categorization. We explored this issue by asking observers to categorize hybrid images immediately following global and local Navon tasks. The composition of hybrid images allows observers to base categorization on either coarse (conveyed by a hybrid's LSFs) or fine (conveyed by a hybrid's HSFs) content. We showed that although observers were sensitive to both types of information (Experiment 1) they overwhelming preferred to base hybrid image categorization on LSF content (Experiments 2–4). When hybrid image categorization was not based on LSF content, HSF-based hybrid image categorization was no better (and often worse) than chance. In Experiment 3, we directly examined how attending to global and local levels of hierarchical Navon figures affected LSF-based hybrid categorization, and found that LSF- based hybrid image categorization was faster following global Navon tasks. This corroborates Flevaris et al. (2011) suggestion that attention to the global level of a hierarchical figure facilitates the selection of LSFs. However, inconsistent with Flevaris and colleagues, control images were all categorized faster following global Navon tasks, suggesting that it was not the priming of absolute spatial frequency *per se* that facilitated LSF-based hybrid image categorization. In Experiment 4, we explored this possibility by replicating Experiment 3 but we forced observers to complete Navon tasks using HSFs, irrespective of the attended level. Similar to Experiment 3, observers preferred to categorize hybrid images based on LSF content. However, and in contrast, LSF-based hybrid image categorization was faster following local Navon tasks, suggesting that LSFs associated with Navon figures were responsible for the scene categorization advantage following global Navon tasks in Experiment 3.

An interesting finding from the present set of studies is that our observers preferred to categorize hybrid images based LSF information in Experiments 2–4, despite the fact that they were sensitive to both spatial frequencies in Experiment 1. One possible explanation is that our masking procedure weakened the signal from HSFs more than the signal from LSFs. Such an explanation suggests that our observers preferred to base hybrid image categorization on the spatial frequency with the strongest signal. Consistent with this hypothesis, Losada and Mullen (1995) showed that white noise masks are more effective at masking HSFs than LSFs. Nevertheless, we regard this possibility as unlikely for two main reasons. First, our observers were more sensitive to a hybrid image's HSFs than LSFs in Experiment 1; and second, as mentioned in the discussion of Experiment 1, our masking effects were particularly weak, suggesting that neither the HSF signal nor the LSF signal were strongly affected by our masking procedure. Our preferred interpretation of these apparent conflicting results is that they corroborate previous research that has shown a critical role for LSFs in rapid scene categorization (Schyns and Oliva, 1994; Oliva and Schyns, 1997; Loschky and Simons, 2004; McCotter et al., 2005). The present results provide further evidence for this hypothesis by demonstrating a preference to use diagnostic LSF information, despite the fact that HSF diagnostic information is more salient.

Another factor possibly affecting the present results is *d*′ values in Experiments 2–4 were calculated by transforming proportion correct as described by Kingdom and Prins (2010). DeCarlo (2012) cautions against using such transformations in m-AFC tasks when the probability of a correct response is greater than 50%, because it assumes that there is no response bias. Although researchers (MacMillan and Creelman, 2005; DeCarlo, 2012) have proposed calculations to control for this potential for response bias, there is currently no accepted procedure in correcting for the bias. Nevertheless, there are several reasons why the transformation used in Experiments 2–4 was unlikely to have affected the interpretation of the present results.

First, the primary purpose of the present set of studies was to investigate spatial scale selection in hybrid images. As previously mentioned, the probability of a correct response on hybrid image trials was 50%, thereby minimizing the response bias concern. Second, although the probability of a correct answer for control image trial types was 25% (for the 4-AFC task), the main purpose of including these results was to demonstrate the availability and sensitivity to different spatial scales in isolation. According to DeCarlo (2012), the largest effect of assuming no response bias is that sensitivity is underestimated. However, since *d*′ values in Experiment 2–4 were above 1.5 for all image types, this suggests that our observers were sensitive to both LSFs and HSFs. Thus, any response bias would only increase the sensitivity measures across the 4-AFC conditions, and thus would not affect interpretation of our results.

A comparison between the present work and the apparent automaticity of scene perception under dual task conditions is particularly relevant. Cohen et al. (2011) suggested that attention task difficulty is the reason some studies have documented impaired scene perception (Walker et al., 2008) whereas others have not (Li et al., 2002; Rousselet et al., 2002). The present work suggests an alternative explanation. Specifically, that impaired scene perception under dual task conditions could be a function of the type of attentional distribution needed to complete the attention task. For example, it seems more likely that a cost of dividing attention would emerge in situations in which the tasks are similar, because the potential for interference from completing the two tasks should be greater. Given that scene categorization was facilitated following global Navon tasks in the present study (at least with unaltered stimuli), suggests the completion of simultaneous attention tasks that require global attention would be more likely to interfere with scene categorization than those that require local attention. Brand et al. (2012) provided support for this hypothesis by demonstrating that the completion of a concurrent task that requires global attention interferes with scene categorization, but a concurrent task that requires local attention does not.

**Figure 9:**
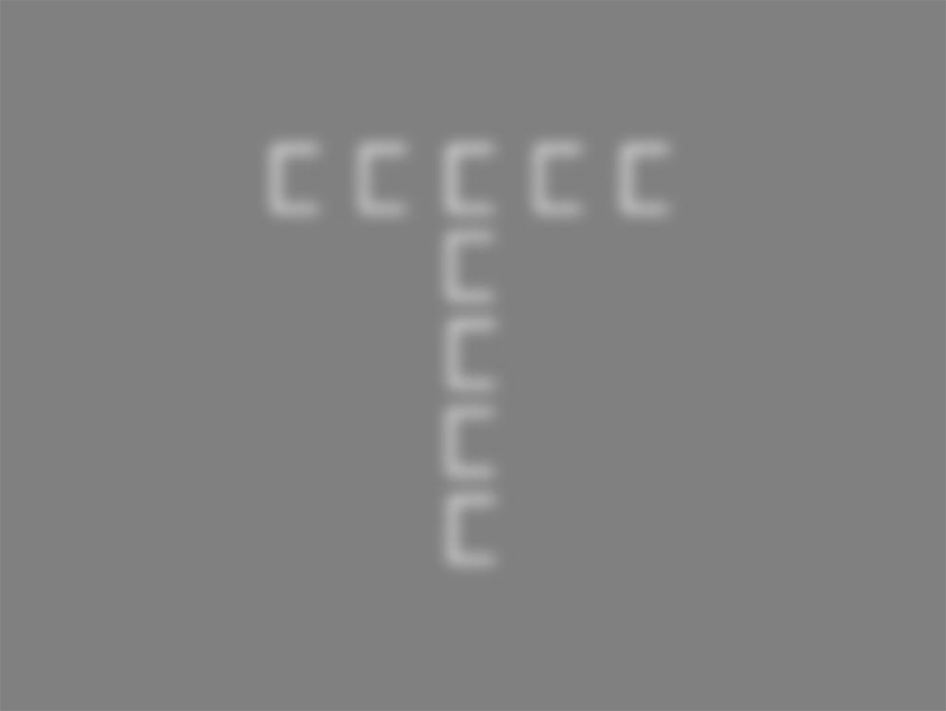
**An example of a low-pass filtered Navon figure**.

One issue the present study was unable to resolve is why scene categorization was faster following local Navon tasks in Experiment 4. This is particularly true for hybrid images, as it is unclear how attending locally would facilitate categorization based on LSF content. If LSFs associated with global Navon tasks facilitated LSF-based hybrid categorization in Experiment 3, then removing that information should have eliminated the global benefit, but should not have resulted in a benefit following local Navon tasks. The fact that it did suggests that observers were using different types of information within a hybrid image's LSF content as the basis for categorization in Experiments 3 and 4, respectively. This conclusion is consistent with Oliva and Schyns' (1997) suggestion that coarse-to-fine information is orthogonal to global-to-local information; that is, there is both coarse and fine information at each spatial scale, and it is possible to direct attention to either level. Consider, for example, the low-pass filtered Navon stimulus in Figure 9. The small “c” represents the image's local features, and the large “T” represents the image's global feature. According to the global-to-local hypothesis, the fine information in the image (i.e., the small c's) should be unrecognizable because the HSFs that convey that information have been removed. Nevertheless, it is evident in the figure that even though HSFs have been removed, that local information remains. Thus, although observers preferred to categorize hybrids based on LSF information in both Experiments 3 and 4, the selection of a Navon's LSFs (Experiment 3) and HSFs (Experiment 4) facilitated the selection of different diagnostic information within a hybrid image's LSF content. Unfortunately, the present study was not designed to identify these differing sources of information.

Another interesting question arising from the present results is whether a hybrid image's HSFs were encoded in Experiments 2–4. Although observers preferred to categorize hybrids based on LSFs in Experiments 2–4, the results of Experiment 1 suggest that both spatial scales were perceptually available. This suggestion is consistent with Oliva and Schyns (1997, Experiments 3 and 4) who showed that when a hybrid image's LSF content is the diagnostic spatial scale, observers nevertheless still process a hybrid image's HSF information implicitly. Along the same lines, de Gardelle and Kouider (2010) found that non-diagnostic spatial scale information could facilitate face perception. de Gardelle and Kouider asked observers to determine whether a full broadband face presented below conscious awareness was of a famous person. A hybrid face preceded the target face and it was constructed such that either its LSFs, or HSFs corresponded with target identify. The critical point here is that face identification is typically based on the relatively HSFs of a hybrid face. Thus, only HSF-hybrid image primes should have facilitated target identification. In contrast, de Gardelle and Kouider reported that both LSF- and HSF-hybrid image primes facilitated target identification. What's more, whereas the effect of HSF-hybrid primes increased significantly with exposure duration, the effect of LSF-hybrid image primes did not. Thus, although LSF information was not diagnostic, it nevertheless played a small role in categorization, most likely restricted to unconscious processing.

The question relating to the role of attention in scene categorization is currently a major source of debate in psychology. Traditionally, this question is addressed by examining the automaticity of scene perception, and whether or not conscious scene perception can occur in the absence of attention. The present article addressed this question from a different angle. It examined how attention facilitates the selection of diagnostic information used in scene categorization. Along the same lines, Larson et al. (2014) showed that manipulations of spatial attention influence the selection of diagnostic scene information. Similar to the global processing bias in the present study, Larson and colleagues reported that scene categorization is initially based on information originating from central vision, with contributions from peripheral vision emerging later on (i.e., a central-to-peripheral processing bias). Larson and colleagues reported that this central processing bias is reduced when the spatial distribution of attention is manipulated so that it emphasizes information in the periphery. Thus, although Larson et al. did not investigate the interaction between attention and spatial scale processing, their results nevertheless converge with the present results to suggest that one role of attention in scene categorization is to select diagnostic scene information.

The primary purpose of the present experiments was to address how attention to local and global levels of Navon figures affects the selection of diagnostic spatial scale information used in scene categorization. This investigation was largely based on the connection between the Navon task spatial scale and the diagnostic spatial scale used for scene categorization. As such, it is reasonable to assume that the categorization of different scene types could also differentially affect the completion of the Navon task. The results of Experiments 3 and 4 allude to this possibility. Whereas Navon processing was slowest when completed in conjunction with low-pass filtered images in Experiment 3, there was no difference in Navon task RTs as a function of scene type in Experiment 4. Although we can only speculate as to the reason for this difference, it appears to be related to the amount of LSFs in the Navon stimuli. Navon stimuli in Experiment 4 were contrast balanced, such that their LSF content was suppressed compared to the Navon stimuli used in Experiment 3. Combined with the fact that LSFs were the preferred diagnostic spatial scale in all experiments, this suggests that the observed Navon slowing in Experiment 3 following low-pass scene categorization was due, in part, to an increased use of LSFs in Experiment 3 compared to Experiment 4.

In conclusion, the present set of experiments demonstrates that attending locally and globally affects the selection of diagnostic spatial scale information used for rapid scene categorization. The present results also converge with previous research in suggesting that LSF information is important in rapid scene categorization (Schyns and Oliva, 1994; Oliva and Schyns, 1997; Loschky and Simons, 2004; McCotter et al., 2005) and extends these findings by demonstrating that the selection of LSF information is affected by manipulations of attention. Thus, although the present results do not conclusively demonstrate that scene perception requires attention, they nevertheless suggest that attention plays a role in facilitating the selection of diagnostic scene information.

### Conflict of interest statement

The authors declare that the research was conducted in the absence of any commercial or financial relationships that could be construed as a potential conflict of interest.

## Supplementary material

Figure S1 **Top:** Log-power spectra for the original and contrast balanced Navon stimuli (averaged over 16 stimuli used in Experiments 3 and 4). In Fourier space, low spatial frequencies are located toward the center of the image, with increasing spatial frequency content toward the image edge. **Bottom:** Log amplitude spectra for stimuli, averaged across orientation, with 95% confidence intervals.

Figure S2 **Example of the original and contrast-balanced Navon stimuli convolved with a bank of log Gabor stimuli of different spatial frequency wavelength (λ, in pixels), with corresponding cycles per image (cpi)**. Color bars represent response of the filter at each spatial frequency, with red depicting a strong response.

## Appendix

### Reduction of low spatial frequency content in contrast balanced navon stimuli

Previous researchers have used contrast balanced Navon stimuli to remove (Lamb and Yund, 1993; Lamb et al., 1999), or reduce (Hübner, 1997; Hübner and Kruse, 2011) the LSFs contained with Navon stimuli. Implementing contrast balanced Navon stimuli encourages observers to use only the remaining HSFs to accomplish both the local and global Navon letter tasks. To verify that the LSF content was reduced in the stimuli used for Experiment 4, we calculated the log-power spectra and rotationally averaged log amplitude spectra of both the contrast balanced and original Navon stimuli.

As can be seen in Figure S1, the balancing of contrast across the edges of local elements of the Navon stimuli has the effect of reducing the overall amplitude at the LSFs, while increasing the amplitude at the HSFs. Therefore, the addition of the borders to the contrast balanced Navon stimuli is not causing a masking effect of the HSFs on the LSFs, as there is a physical reduction in the LSF content.

One possible explanation for this reduction in LSF content is due to the Fourier analysis introducing an artifact into the stimuli. To exclude this possibility, we convolved the stimuli with a bank of log Gabor filters in the spatial domain. Log Gabor filters were created in Mathworks Matlab (ver. 2013b), using a starting minimum wavelength of the filter to be 16 pixels (or 46.9 cpi). Each filter was rendered at 6 possible orientations (0–150° in 30° increments), with the final response at each spatial frequency being created by averaging across all orientations. Each subsequently LSF filter doubled the wavelength, creating a total of four spatial frequencies (46.9, 23.4, 11.7, 5.9 cpi). As can be seen in Figure S2, at the highest spatial frequency (46.9 cpi), the contrast balanced Navon stimuli show a stronger response relative to the original Navon stimuli. However at LSFs (23.4, 11.7, and 5.9 cpi), the contrast balanced Navon stimuli show a weaker response in comparison to the original Navon stimuli. We therefore conclude that the reduction of the LSF component introduced by the contrast balanced Navon is not an artifact, but instead represents a quantifiable reduction in LSF content.
