# Supplementary figures and images for: Attention to local and global levels of hierarchical Navon figures affects rapid scene categorization
Source: Front Psychol. 2014 Dec 2;5:1274. doi: 10.3389/fpsyg.2014.01274 (PMC4251296; doi:10.3389/fpsyg.2014.01274)

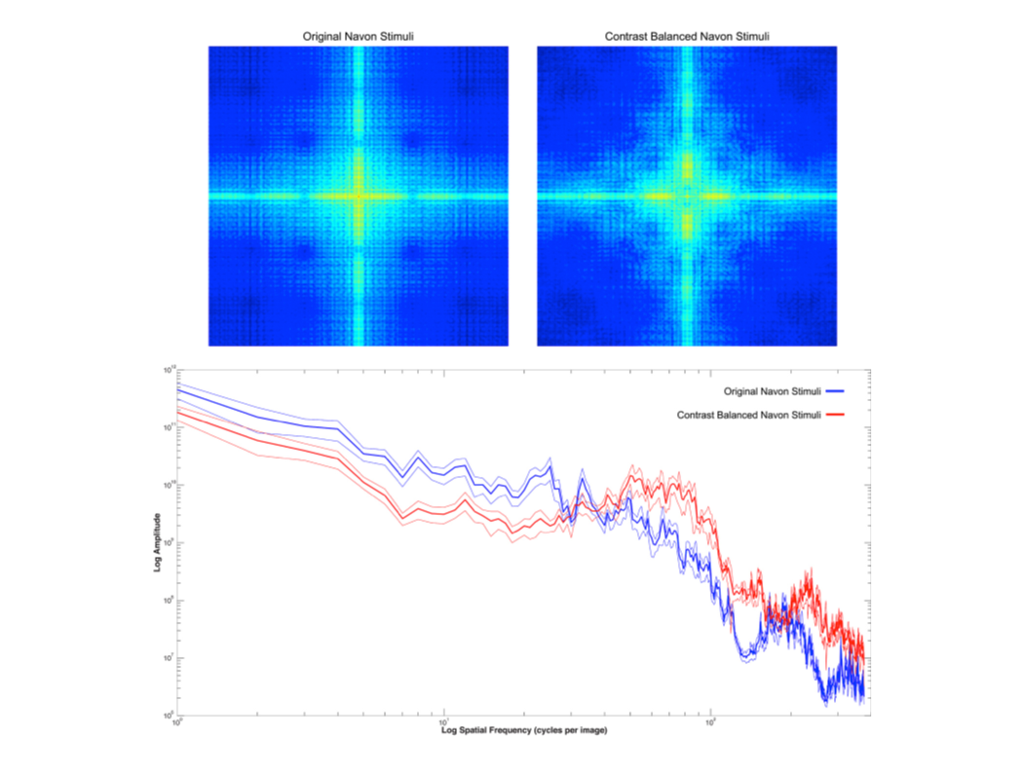

Supplement: Figure S1 — Top: Log-power spectra for the original and contrast balanced Navon stimuli (averaged over 16 stimuli used in Experiments 3 and 4). In Fourier space, low spatial frequencies are located toward the center of the image, with increasing spatial frequency content toward the image edge. Bottom: Log amplitude spectra for stimuli, averaged across orientation, with 95% confidence intervals. [file Image1.TIFF]

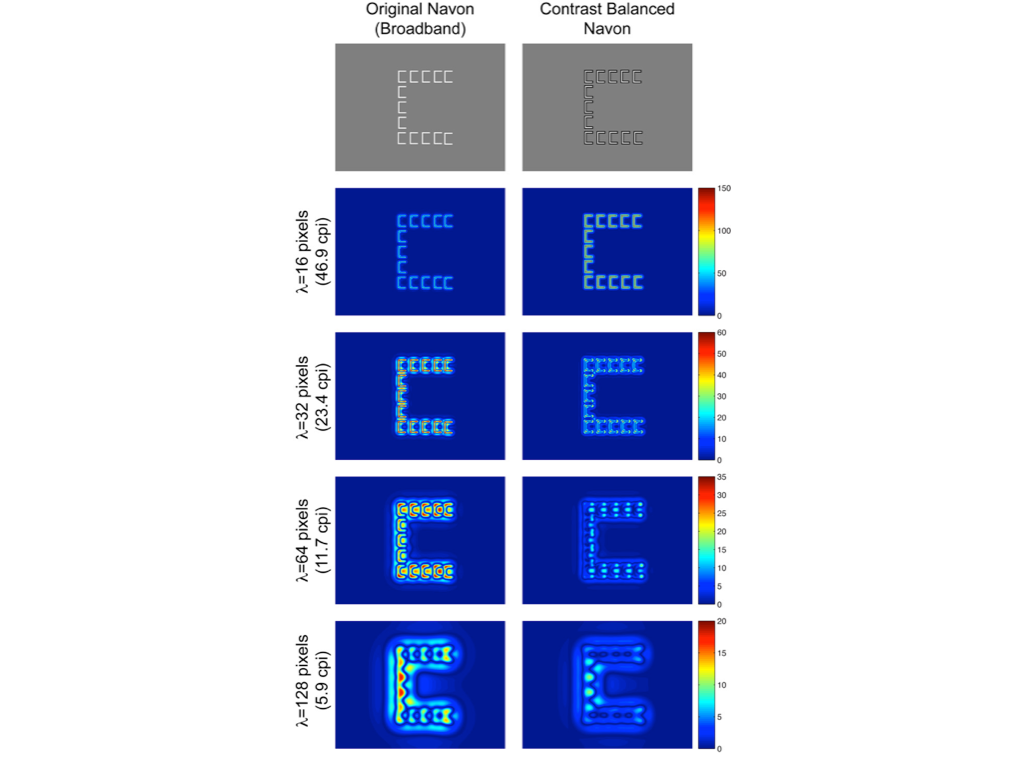

Supplement: Figure S2 — Example of the original and contrast-balanced Navon stimuli convolved with a bank of log Gabor stimuli of different spatial frequency wavelength (λ, in pixels), with corresponding cycles per image (cpi). Color bars represent response of the filter at each spatial frequency, with red depicting a strong response. [file Image2.TIFF]
